# Clarifying and extending permutation tests on brain map correspondence through mixed-effects modeling

**DOI:** 10.1162/IMAG.a.1350

**Published:** 2026-09-08

**Authors:** Qiaochu Wang, Lingyi Peng, Thomas E. Nichols, Xu Zou, Yaotian Wang, Jie He, Yuexin Zhang, Dana L. Tudorascu, Diego Szczupak, Lauren Schaeffer, Emily S. Rothwell, Laurel Dieckhaus, Stacey J. Sukoff Rizzo, Gregory W. Carter, Afonso C. Silva, Tingting Zhang

**Affiliations:** Department of Statistics, University of Pittsburgh, Pittsburgh, PA, United States; Department of Biostatistics and Health Data Science, University of Pittsburgh, Pittsburgh, PA, United States; Oxford Big Data Institute, Nuffield Department of Population Health, University of Oxford, Oxford, United Kingdom; Department of Biostatistics and Bioinformatics, Emory University, Atlanta, GA, United States; Department of Neurobiology, University of Pittsburgh, Pittsburgh, PA, United States; The Jackson Laboratory, Bar Harbor, ME, United States

**Keywords:** brain map correspondence, mixed-effects models, permutation tests, bootstrapping

## Abstract

Permutation-based methods such as the spin test, Brain Surrogate Maps with Autocorrelated Spatial Heterogeneity (BrainSMASH), and the simple permutation-based intermodal correspondence (SPICE) test are widely used to assess correspondence between brain maps while accounting for their spatial autocorrelation. However, these methods define and evaluate correspondence in fundamentally different ways, making their results difficult to compare or interpret jointly. We address these limitations by introducing a two-factor mixed-effects model that decomposes brain map variability into components arising from inter-subject variability and spatial variability across brain locations. This formulation provides a principled way to characterize distinct sources of variability in brain maps and to formally link each correspondence test to the specific component it targets. Within this framework, we further provide the analytical expressions of the null distributions of the spin test, BrainSMASH, and SPICE in terms of model parameters. This unified framework clarifies the fundamental distinctions among the permutation tests, reveals their implicit assumptions, and provides a principled way to compare and interpret their results. Beyond clarifying existing methods, the modeling framework naturally motivates a bootstrap-based method that enables simultaneous inference of multiple forms of correspondence arising from different components of brain map variability. Through extensive simulations and empirical analyses of both structural (cortical thickness vs. sulcal depth) and functional (language vs. motor contrast) brain maps, we demonstrate that the bootstrap-based method achieves well-calibrated type I error, substantially higher statistical power, and provides a robust and comprehensive characterization of different forms of brain map correspondence.

## 1 Introduction

Recent advances in neuroimaging, brain tracing, and high-resolution recording technologies have dramatically expanded our capacity to observe and analyze the human brain (Insel et al., 2013). These technological breakthroughs have led to the generation of enormous datasets in various modalities that capture various aspects of brain organization. These include multidimensional genomic and transcriptomic data (Akbarian et al., 2015; Hansen et al., 2021), cellular architecture and cytoarchitecture (von Economo & Koskinas, 1925), neurotransmitter receptor maps (Hansen et al., 2022; Nørgaard et al., 2021), metabolic activity (Vaishnavi et al., 2010), and various imaging modalities on brain functional and structural features (Markello et al., 2022; Yen et al., 2023), such as EEG, MEG (Darvas et al., 2004), magnetic resonance imaging (MRI), functional MRI (fMRI), computed tomography (CT), and positron emission tomography (PET) (Rogers et al., 2007; Toga & Mazziotta, 2002; Toga & Thompson, 2001; Yeo et al., 2011).

With the rapid expansion of multimodal brain datasets, there is growing interest in comparing spatially distributed brain features and quantifying their association, also called correspondence, to reveal the biological mechanisms and organizational principles underlying brain structure and function (Baum et al., 2020; Gao et al., 2020; Hansen et al., 2021; Vázquez-Rodríguez et al., 2019). This scientific need has motivated the development of a wide range of statistical methods for evaluating correspondence between brain maps. Standard approaches include correlation-based similarity measures (Horn et al., 2014; Smith et al., 2009) and regression-based analysis (Mandal et al., 2020; Miri et al., 2011). However, conventional approaches typically ignore the strong spatial autocorrelation inherent in brain maps. Failure to account for this property can lead to inflated false positive or false negative rates (Eklund et al., 2016; Markello & Misic, 2021; Mueller et al., 2017; Slotnick, 2017).

To address these concerns, spatially constrained permutation tests have been widely adopted to generate more appropriate null models for correspondence analyses. These methods preserve the spatial autocorrelation structure of brain maps while disrupting their spatial alignment. Specifically, the spin test (Alexander-Bloch et al., 2018) produces the null model by rotating spherical surfaces, thereby preserving spatial dependencies in brain maps. Brain Surrogate Maps with Autocorrelated Spatial Heterogeneity (BrainSMASH) (Burt et al., 2020) generate surrogate maps that match the empirical spatial autocorrelation of a target map. The simple permutation-based intermodal correspondence (SPICE) test (Weinstein et al., 2021) uses average correlations between randomly paired subjects’ brain maps to build the null distribution. These approaches have become standard tools due to their conceptual simplicity and their ability to account for spatial autocorrelation structures of brain maps.

Despite their widespread use, these permutation tests suffer from two key limitations. First, they do not share a consistent or unified definition of correspondence. Although all methods compare an observed statistic with a permutation-based null distribution, the test statistics and permutation procedures differ fundamentally. For example, the spin test assesses the significance of correspondence by comparing the correlation between group-average brain maps to an empirical distribution generated from randomly rotated group-average maps. In contrast, the SPICE test compares the average correlation between individual subjects’ brain maps with the average correlations computed from randomly paired subjects’ maps. As a result, it remains unclear how the results from different tests relate to one another or how they should be compared, despite some empirical comparisons such as those by Markello and Misic (2021). Second, permutation tests rarely specify explicit alternative hypotheses. Although a rejection indicates that the observed statistic deviates from the null distribution, these methods do not reveal what features of brain maps drive this deviation or to what extent different components of brain map variability contribute to the observed correspondence. Consequently, existing approaches provide only a partial understanding of relationships among multimodal brain maps. As noted by Markello and Misic (2021), there remains a strong need for rigorous statistical frameworks for assessing brain map correspondence.

In this work, we address these limitations by introducing two-factor mixed-effects models for brain maps. Extending recent applications of a similar model to brain networks (Peng et al., 2025), we adapt this model to brain maps by decomposing their variability into separate interpretable components: inter-subject variability and spatial variability across brain locations. This formulation establishes a direct connection between scientifically meaningful brain map features and statistical model parameters. Crucially, we show that the null distributions underlying the spin test, BrainSMASH, and the SPICE test can be expressed analytically in terms of these model parameters. This unified perspective clarifies the fundamental distinctions among permutation tests, reveals their implicit assumptions, and provides a principled way to compare their results and interpret their behavior across diverse correspondence scenarios.

Beyond clarifying existing methods, the modeling framework naturally motivates a bootstrap-based method for inferring brain map correspondence. Unlike any single permutation test, this model-driven procedure exploits the full structure of the mixed-effects formulation, enabling inference on distinct correspondence forms attributable to specific sources of brain map variability. Extensive simulation studies and real data analyses demonstrate that the bootstrap-based method provides competitive type I error control and statistical power under the assumptions of existing methods, while offering greater flexibility, interpretability, and applicability to scenarios not well handled by any individual permutation test.

## 2 Materials and Methods

### 2.1 Data acquisition

Neuroimaging data under analysis include structural MRI and task-based functional MRI (tfMRI) data of 1,206 healthy young adults from the Human Connectome Project (HCP) S1200 release. All scans were acquired on a customized 3T Siemens scanner. High-resolution T1- and T2-weighted structural images were collected at 0.7 mm isotropic resolution, while tfMRI data were acquired with 2 mm isotropic voxels and a temporal resolution of 720 ms using a multiband echo-planar imaging (EPI) sequence (Feinberg et al., 2010; Glasser et al., 2013; Moeller et al., 2010; Uğurbil et al., 2013).

We implemented quality control (QC) following the standard HCP protocol (Marcus et al., 2013), excluding participants with anatomical anomalies, segmentation or surface reconstruction errors, or acquisition instabilities. After this step, 1,060 participants remained. To ensure consistency across imaging modalities, we further restricted analyses to individuals with both high-quality structural MRI and tfMRI datasets, resulting in a final cohort of 900 participants.

### 2.2 Imaging preprocessing

All imaging data underwent the HCP minimal preprocessing pipelines (Glasser et al., 2013), which corrected for spatial distortions, performed motion realignment, and registered data to the fs_LR 32k cortical surfaces under the CIFTI grayordinate framework. The preprocessing steps produced cortical surfaces with approximately 32,000 vertices per hemisphere. The HCP data under analysis were also registered with a multimodal surface registration algorithm that uses areal features, including myelin maps and resting-state networks, to improve cross-subject alignment of cortical areas (Robinson et al., 2018, 2014). For all analyses, we restricted data to the cortical surface, excluding the medial wall.

### 2.3 Brain map generation

**Fig. 1.**
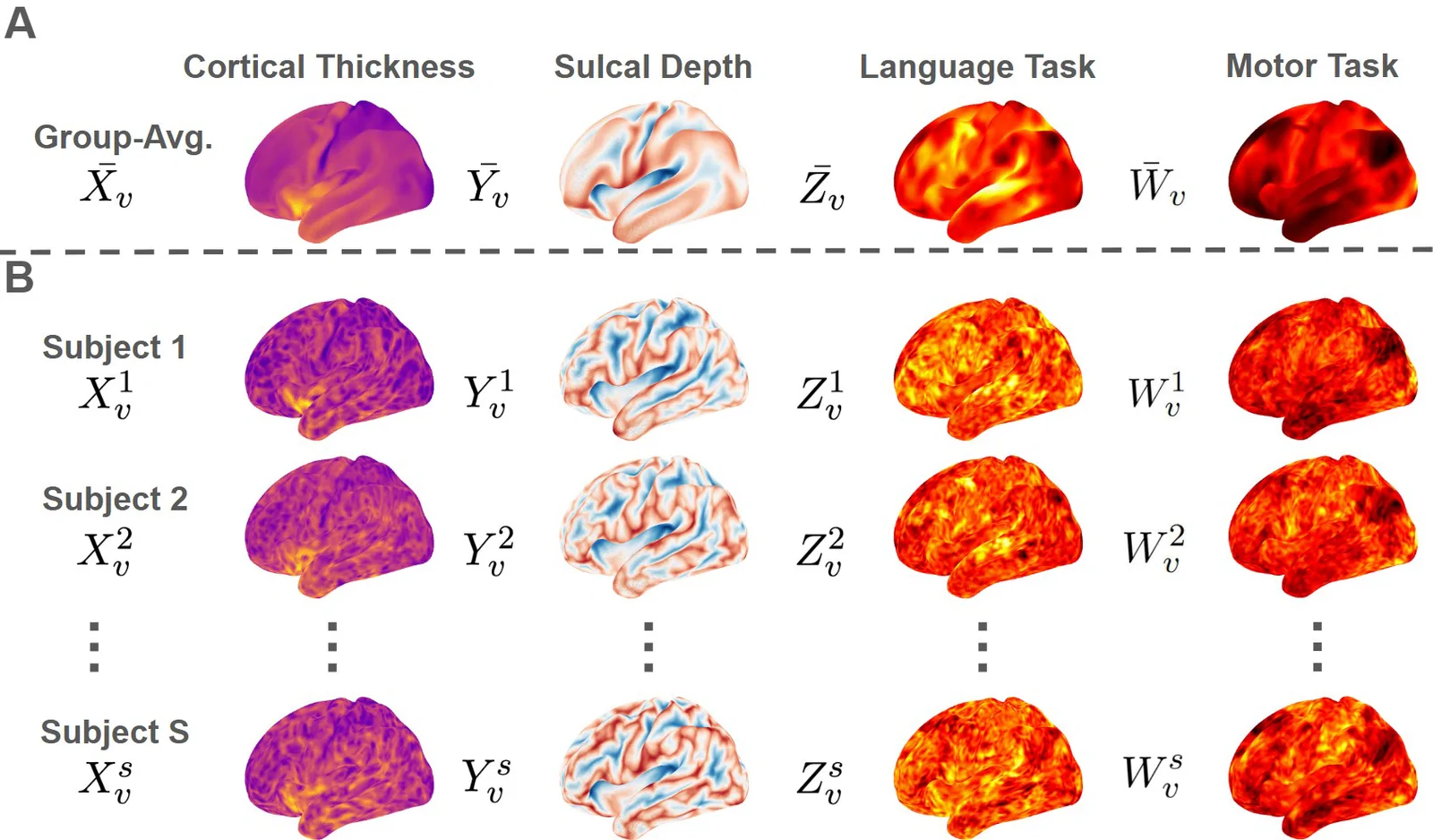
Group-average and subject-level brain maps. (A) Group-average brain maps from 900 subjects for cortical thickness, sulcal depth, the language contrast, and the motor contrast. (B) Subject-level brain maps for the same four modalities. All maps are shown at the vertex level for the left hemisphere in a lateral view.

We analyzed four types of subject-level brain maps directly available from the HCP S1200 release (Fig. 1B), including two structural maps, cortical thickness, and sulcal depth derived from structural MRI, and two functional maps derived from task-based fMRI data.

Specifically, we used fMRI data from two task contrasts: the story–math contrast and the movement contrast. The former contrast compares brain activity during an auditory story comprehension condition with that during an arithmetic condition, capturing regions involved in language processing. The movement contrast captures the average activation across multiple movement conditions (left hand, right hand, left foot, right foot, and tongue) relative to fixation, capturing motor-related brain activity. For simplicity, we refer to these as the language task and motor task brain maps, respectively.

The functional maps were obtained using the standard HCP task-fMRI general linear model (GLM) pipeline (Barch et al., 2013). Briefly, for each subject, task-related activation was estimated using a GLM with regressors corresponding to experimental conditions convolved with the canonical hemodynamic response function, along with nuisance regressors (e.g., motion parameters). The resulting statistical maps were provided in the HCP grayordinate CIFTI format, and we used the vertex-wise *z*-statistics from the released contrast maps.

For each subject, we analyzed each type of brain map at three spatial resolutions: a dense vertex-level map and two parcel-level maps. The vertex-level maps were obtained directly from the HCP minimal preprocessing pipelines and defined on the fs_LR 32k cortical surface within the CIFTI grayordinate framework. We created parcel-level maps by parcellating the vertex-level brain maps using Connectome Workbench software (Marcus et al., 2011). Specifically, we applied two widely used atlases to generate the maps: the HCP Multi-Modal Parcellation (MMP1.0), which defines 360 cortical areas (Glasser et al., 2016), and the Schaefer parcellation (Kong et al., 2021; Schaefer et al., 2018). For each parcellation, the value at each parcel was computed as the average of vertex values within the parcel. Additional methodological and validation details on these multi-resolution maps are provided in Supplementary Section S1, and group-average maps at all three spatial resolutions are shown in Supplementary Figure S1.

In summary, for each subject, we analyzed four types of brain maps: cortical thickness, sulcal depth, language contrast, and motor contrast. Each of these brain maps was represented at three spatial resolutions (vertex, 360 parcels, and 1,000 parcels). These multi-resolution maps were used in all subsequent analyses.

### 2.4 Mixed-effects models

Let $X_{v}^{s}$ and $Y_{v}^{s}$ denote the observed values at brain location *v* in two brain maps *X* and *Y* of subject *s*, where v=1, …, V indexes brain locations and s=1, …,S indexes subjects. Here *V* and *S* denote, respectively, the total number of brain locations and subjects in the study. We consider the *V* brain locations to be fixed given the brain parcellation, while *S* subjects are randomly sampled from the population. Therefore, the model parameters associated only with brain location *v* are considered fixed while parameters associated with subject *s* are random.

We propose the following mixed-effects models for $X_{v}^{s}$ and $Y_{v}^{s}$:

$$X_{v}^{s}\;=\mu_{X}\;+\alpha_{X,v}\;+\beta_{X}^{s}\;+\eta_{X,v}\omega_{X}^{s}\;+\epsilon_{X,v}^{s},$$ (1)

$$Y_{v}^{s}\;=\;\mu_{Y}\;+\alpha_{Y,v}\;+\beta_{Y}^{s}\;+\eta_{Y,v}\omega_{Y}^{s}\;+\epsilon_{Y,v}^{s},$$ (2)

where *μ_X_* and *μ_Y_* are fixed constants representing the global means of *X* and *Y* brain maps across all locations and the entire subject population. The other components capture different sources of variability:

- Main spatial effects α_X, *v*_ and α_Y, *v*_. These fixed effects represent population-mean location-specific deviations from the global means. In particular, the values at location *v* in the population-mean *X* and *Y* brain maps are given by
  $$\begin{array}{l} \mu_{X}+\alpha_{X,v}\;=E\left(X_{v}^{s}\right)=\underset{S\to \infty }{\text{lim}}\frac{1}{S}\sum_{s=1}^{S}X_{v}^{s}\;\text{and}\\ \mu_{Y}+\alpha_{Y,v}=E\left(Y_{v}^{s}\right)=\underset{S\to \infty }{\text{lim}}\frac{1}{S}\sum_{s=1}^{S}Y_{v}^{s}, \end{array}$$ (3)
  where 𝔼(·) denotes the expectation over the population of all subjects. The main spatial effects capture spatial variability in population-mean brain maps.
- Main subject effects $\beta_{X}^{s}$ and $\beta_{Y}^{s}$. These random effects represent subject-specific whole-brain shifts relative to the population-mean *X* and *Y* brain maps. Specifically,
  $$\begin{array}{l} \frac{1}{V}\sum_{v=1}^{V}X_{v}^{s}=\frac{1}{V}\sum_{v=1}^{V}\left(\mu_{X}+\alpha_{X,v}\right)+\beta_{X}^{s}\;\text{and}\;\\ \frac{1}{V}\sum_{v=1}^{V}Y_{v}^{s}=\frac{1}{V}\sum_{v=1}^{V}\left(\mu_{Y}+\alpha_{Y,v}\right)+\beta_{Y}^{s}. \end{array}$$
  Random subject-specific $\beta_{X}^{s}$ and $\beta_{Y}^{s}$, respectively, capture whether subject *s* has overall “higher” or “lower” *X* and *Y* brain maps across the entire brain relative to the population-mean brain maps. Therefore, main subject effects quantify between-subject variability in the brain-wide average.
- Interaction effects $\eta_{X,v}\omega_{X}^{s}$ and $\eta_{Y,v}\omega_{Y}^{s}$: These product terms characterize subject-specific spatially structured deviations from the population-mean *X* and *Y* maps. Specifically, the spatial components ${\left\{\eta_{X,v}\right\}}_{v=1}^{V}$ and ${\left\{\eta_{Y,v}\right\}}_{v=1}^{V}$ are fixed and represent the dominant spatial patterns of variation shared across subjects, that is, systematic spatial variation in which individual subjects’ brain maps deviate most from their population means. The coefficients $\omega_{X}^{s}$ and $\omega_{Y}^{s}$ are subject-specific random effects that quantify how strongly brain maps of subject *s* express these patterns. We refer to η_X, *v*_ and η_Y, *v*_ as interaction spatial effects in *X* and *Y* brain maps, respectively, while $\omega_{X}^{s}$ and $\omega_{Y}^{s}$ as interaction subject effects in *X* and *Y* brain maps.
- Residuals $\epsilon_{X,v}^{s}$ and $\epsilon_{Y,v}^{s}$. Random residuals represent the remaining variability in subject *s*’ *X* and *Y* brain maps not accounted for by the main or interaction effects. These two terms capture location and subject-specific fluctuations that are not aligned with population-mean patterns or the dominant systematic spatial variation.

**Fig. 2.**
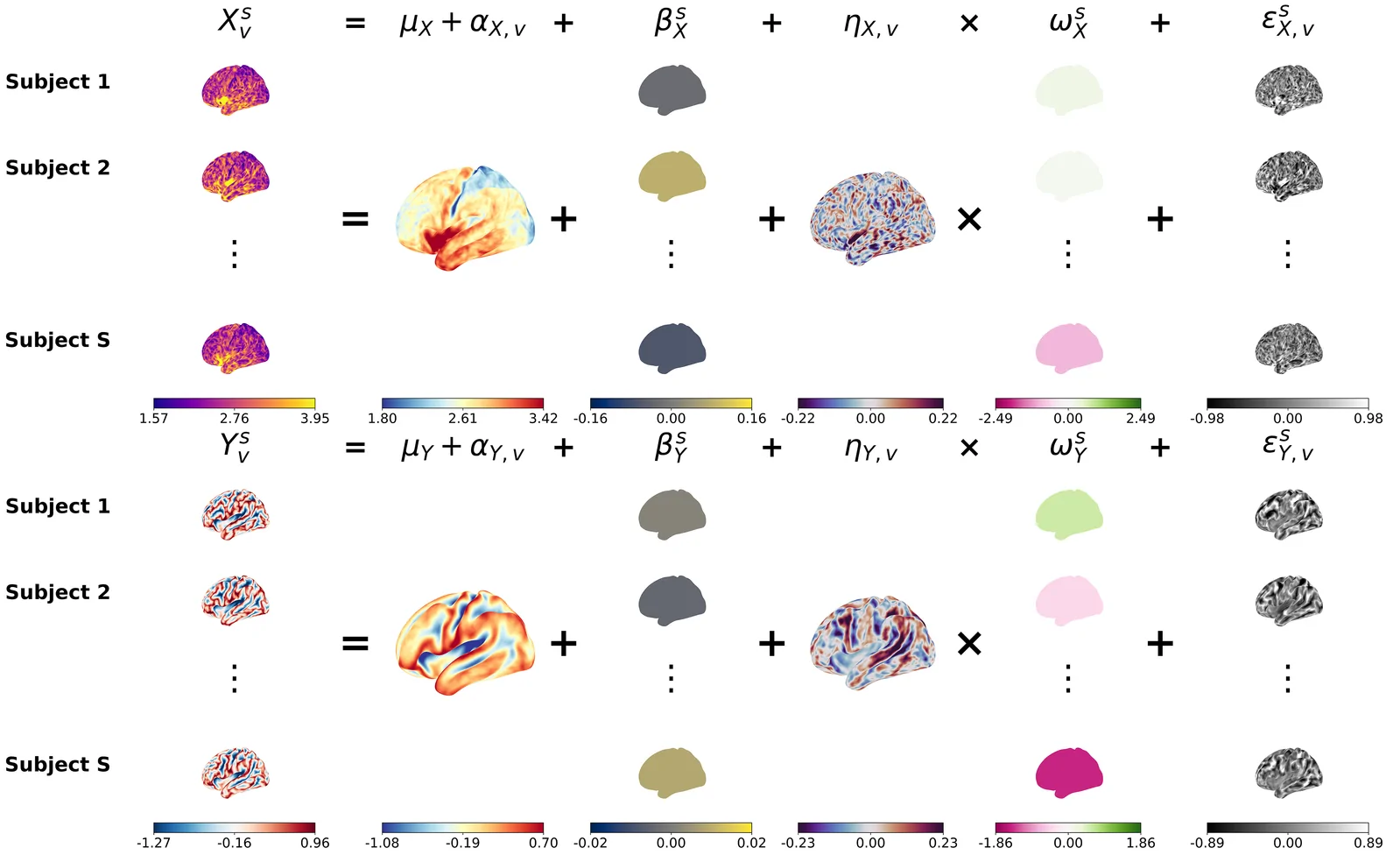
Visualization of the mixed-effects models for brain maps, which consist of four components: (1) the population-mean map (e.g., $\mu_{X}+\alpha_{X,v}$ ), shown as a single spatial pattern shared across all subjects; (2) the main subject effect ($\beta_{X}^{s}$), depicted as subject-specific scalar offsets (solid colors); (3) the interaction effect ($\eta_{X,v}\cdot \;\omega_{X}^{s}$), visualized as a fixed spatial map multiplied by subject-specific scaling factors; and (4) the residual term ($\epsilon_{X,v}^{s}$), represented as grayscale noise maps. Color bars indicate the value range for each column.

Figure 2 provides a schematic visualization of the proposed mixed-effects models, illustrating how fixed spatial effects and random subject effects together constitute brain maps.

To avoid identifiability issues between the global means and the spatial effects, we impose centering constraints on the fixed spatial effects:

$$\sum_{v=1}^{V}\alpha_{X,v}\;=\sum_{v=1}^{V}\alpha_{Y,v}\;=\sum_{v=1}^{V}\eta_{X,v}\;=\sum_{v=1}^{V}\eta_{Y,v}\;=0.$$ (4)

We allow for flexible dependence among subject-level random effects and residuals. For each subject *s*, denote the associated random variables by

$$R^{s}:={\left(\beta_{X}^{s},\;\beta_{Y}^{s},\;\omega_{X}^{s},\;\omega_{Y}^{s},\;\epsilon_{X,1}^{s},\;\epsilon_{Y,1}^{s},\;\ldots ,\;\epsilon_{X,V}^{s},\;\epsilon_{Y,V}^{s}\right)}^{⊤}.$$

We assume that $\left(R^{1},\;\ldots ,\;R^{S}\right)$ are independent and identically distributed (i.i.d.) with zero means.

Finally, to ensure identifiability of the interaction terms, we fix the variances of interaction subject effects to one: $\text{Var}\left(\omega_{X}^{s}\right)=\text{Var}\left(\omega_{Y}^{s}\right)=1$ , where Var(·) represents the variance calculated across all subjects in the population. For example, $\text{Var}\left(\omega_{X}^{s}\right)={\underset{S\to \infty }{\text{lim}}}_{s=1}^{S}{\left(\omega_{X}^{s}\right)}^{2}/S=1$ .

Table 1 summarizes the notations for the sets, constants, fixed effects, random effects, and derived quantities used in the mixed-effects models. Table 2 provides the notations for statistical operators, correlations, and their corresponding estimators that appear in the subsequent analytical derivations for the permutation tests.

**Table 1.** Notation for sets, constants, model parameters, and derived quantities.

Notations	Description	Type
***Sets and Constants***		
*V*	The total number of brain locations	Constant
*S*	The total number of subjects in the data	Constant
*V*	The set of all brain locations {1, …, V}	Set
*S*	The set of all subjects in the data {1, …, S}	Set
**Model Parameters: Fixed Effects**		
*μ_X_*, *μ_Y_*	Global means for *X* and *Y*	Fixed
α_*X*, *v*_, α_*Y*, *v*_, v∈V	Main spatial effects for *X* and *Y* brain maps	Fixed
η_*X*, *v*_, η_*Y*, *v*_, v∈V	Interaction spatial effects for *X* and *Y* brain maps	Fixed
**Model Parameters: Random Variables**		
$\beta_{X}^{s}$, $\beta_{Y}^{s}$, s∈S	Main subject effects for *X* and *Y* brain maps	Random
$\omega_{X}^{s}$, $\omega_{Y}^{s}$, s∈S	Interaction subject effects for *X* and *Y* brain maps	Random
$\epsilon_{X,v}^{s}$, $\epsilon_{Y,v}^{s}$, v∈V and s∈S	Residual terms	Random
**Derived Quantities**		
$\hat{\theta }$ (e.g., $\hat{\rho }$ , ${\hat{\alpha }}_{X,v}$ )	Empirical estimate of a parameter θ	Random
Ā (e.g., ${\bar{X}}_{v}$, ${\bar{\beta }}_{X}$)	Average of *A^s^* over subjects s∈S	Random
$\tilde{A}$ (e.g., ${\tilde{\alpha }}_{X}$, ${\tilde{\eta }}_{X}$)	Average of *A_v_* over locations v∈V	Fixed/Random

**Table 2.** Statistical operators, correlations, and their estimators.

Symbol	Description	Example/Definition
**Statistical Operators Performed over the Entire Subject Population**		
Corr(·,·)	Correlation operator across all subjects in the population	e.g., $\text{Corr}\left(\beta_{X}^{s},\;\beta_{Y}^{s}\right)$
Var(·)	Variance operator across all subjects in the population	e.g., $\text{Var}\left(\omega_{X}^{s}\right)$
𝔼(·)	Expectation operator across all subjects in the population	e.g., $E\left(X_{v}^{s}\right)$, $E\left[{\text{Corr}}_{V}\left(X_{v}^{k_{s}},\;Y_{v}^{s}\right)\right]$
**Statistical Operators Performed on Finite Sets** V **or** S		
${\text{Corr}}_{V}\left(\cdot ,\cdot \right)$	Correlation operator across locations v∈V	e.g., ${\text{Corr}}_{V}\left({\bar{X}}_{v},{\bar{Y}}_{v}\right)$, ${\text{Corr}}_{V}\left(\alpha_{X,v},\;\alpha_{Y,v}\right)$
${\text{Cov}}_{V}\left(\cdot ,\cdot \right)$	Covariance operator across locations v∈V	e.g., ${\text{Cov}}_{V}\left(\alpha_{X,\pi \left(v\right)},\;\alpha_{Y,v}\right)$
${\text{Var}}_{V}\left(\cdot \right)$	Variance operator across locations v∈V	e.g., ${\text{Var}}_{V}\left(\alpha_{X,v}\right)$
${\text{Corr}}_{S}\left(\cdot ,\cdot \right)$	Correlation operator across subjects s∈S	e.g., ${\text{Corr}}_{S}\left({\hat{\beta }}_{X}^{s},\;{\hat{\beta }}_{Y}^{s}\right)$
	$\underset{S\to \infty }{\text{lim}}{\text{Corr}}_{S}\left(\cdot ,\cdot \right)=\text{Corr}\left(\cdot ,\cdot \right)$	e.g., $\underset{S\to \infty }{\text{lim}}{\text{Corr}}_{S}\left(\beta_{X}^{s},\;\beta_{Y}^{s}\right)=\text{Corr}\left(\beta_{X}^{s},\;\beta_{Y}^{s}\right)$
**Correlation Parameters (Targets)**		
*ρ*	Correlation between population-mean brain	${\text{Corr}}_{V}\left(\mu_{X}+\alpha_{X,v},\;\mu_{Y}+\alpha_{Y,v}\right)$
	maps (i.e., between main spatial effects)	$={\text{Corr}}_{V}\left(\alpha_{X,v},\;\alpha_{Y,v}\right)$
ρ_β, β_	Correlation between main subject effects	$\text{Corr}\left(\beta_{X}^{s},\;\beta_{Y}^{s}\right)$
ρ_ω, ω_	Correlation between interaction subject effects	$\text{Corr}\left(\omega_{X}^{s},\omega_{Y}^{s}\right)$
ρ_ϵ, ϵ_	Average correlation between residuals	$\Sigma_{v=1}^{V}\text{Corr}\left(\in_{X,v}^{s},\in_{Y,v}^{s}\right)/V$
ρ_ϵ, ω_	Average correlation between residuals and interaction effects	$\Sigma_{v=1}^{V}\text{Corr}\left(\in_{X,v}^{s},\omega_{Y}^{s}\right)/V$
ρ_ω, ϵ_	Average correlation between interaction effects and residuals	$\sum_{v=1}^{V}\text{Corr}\left(\omega_{X}^{s},\;\epsilon_{Y,v}^{s}\right)/V$
**Empirical Correlation Estimators (Statistics)**		
$\hat{\rho }$	Estimator of ρ	${\text{Corr}}_{V}\left({\bar{X}}_{v},\;{\bar{Y}}_{v}\right)$
${\hat{\rho }}_{\beta ,\beta }$	Estimator of ρ_β, β_	${\text{Corr}}_{S}\left({\hat{\beta }}_{X}^{s},\;{\hat{\beta }}_{Y}^{s}\right)$
${\hat{\rho }}_{\omega ,\omega }$	Estimator of ρ_ω, ω_	${\text{Corr}}_{S}\left({\hat{\omega }}_{X}^{s},{\hat{\omega }}_{Y}^{s}\right)$
${\hat{\rho }}_{\epsilon ,\epsilon }$	Estimator of ρ_ϵ, ϵ_	$\sum_{v=1}^{V}{\text{Corr}}_{S}\left({\hat{\epsilon }}_{X,v}^{s},\;{\hat{\epsilon }}_{Y,v}^{s}\right)/V$
${\hat{\rho }}_{\epsilon ,\omega }$	Estimator of ρ_ϵ, ω_	$\Sigma_{v=1}^{V}{\text{Corr}}_{S}\left({\hat{\in }}_{X,v}^{s},{\hat{\in }}_{Y,v}^{s}\right)/V$
${\hat{\rho }}_{\omega ,\epsilon }$	Estimator of ρ_ω, ϵ_	$\sum_{v=1}^{V}{\text{Corr}}_{S}\left({\hat{\omega }}_{X}^{s},{\hat{\epsilon }}_{Y,v}^{s}\right)/V$
**Expectation Operators Performed Across Permutations**		
𝔼*_π_*(·)	Expectation operator across all spin rotations *π*	e.g., 𝔼*_π_*[α_*X*, *π*(*v*)_]
𝔼*_P_*(·)	Expectation operator across all BrainSMASH permutations *P*	e.g., $E_{P}\left[{\text{Cov}}_{V}\left(\sum w_{v,P\left(j\right)}\alpha_{X,j},\;\alpha_{Y,v}\right)\right]$

### 2.5 Permutation tests under mixed-effects models

We parameterize the null hypotheses of permutation tests, including the spin test, BrainSMASH, and the SPICE test, within the mixed-effects modeling framework (1) and (2). By analytically comparing each test statistic with its corresponding permutation statistics, we derive the precise parameter conditions under which the observed statistic follows the same distribution (i.e., the null distribution or null model) as its permutation statistics. This formulation explicitly delineates the null hypotheses of these tests based on the model parameters and provides a coherent basis for understanding their assumptions and comparing their inferential properties within a common theoretical framework.

#### 2.5.1 Correlation between group-average brain maps

A commonly used measure of correspondence between two brain maps is the Pearson correlation between their group-average values across all brain locations. This correlation is also used as the test statistic in both the spin test and BrainSMASH. Under the mixed-effects models (1) and (2), the group-average *X* and *Y* brain maps are

$$\begin{array}{l} {\bar{X}}_{v}\;=\mu_{X}\;+\alpha_{X,v}\;+{\bar{\beta }}_{X}\;+\eta_{X,v}{\bar{\omega }}_{X}+{\bar{\epsilon }}_{X,v}\text{ and}\;\\ {\bar{Y}}_{v}=\mu_{Y}+\alpha_{Y,v}+{\bar{\beta }}_{Y}+\eta_{Y,v}{\bar{\omega }}_{Y}+{\bar{\epsilon }}_{Y,v}, \end{array}$$

where ${\bar{\beta }}_{X}=\sum_{s=1}^{S}\beta_{X}^{s}/S$ , ${\bar{\beta }}_{Y}\;=\sum_{s=1}^{S}\beta_{Y}^{s}/S$ , ${\bar{\omega }}_{X}\;=\sum_{s=1}^{S}\omega_{X}^{s}/S$ , ${\bar{\omega }}_{Y}\;=\sum_{s=1}^{S}\omega_{Y}^{s}/S$ , ${\bar{\epsilon }}_{X,v}\;=\sum_{s=1}^{S}\epsilon_{X,v}^{s}/S$ , and ${\bar{\epsilon }}_{Y,v}\;=\sum_{s=1}^{S}\epsilon_{Y,v}^{s}/S$ . The overbar notation (e.g., ${\bar{\beta }}_{X}$) denotes an average across subjects s∈S={1, …, S}.

The Pearson correlation between the group-average *X* and *Y* brain maps is given by

$${\text{Corr}}_{V}\left({\bar{X}}_{v},{\bar{Y}}_{v}\right)=\frac{\begin{array}{l} \sum_{v=1}^{V}\left[\alpha_{X,v}+\eta_{X,v}{\bar{\omega }}_{X}+{\bar{\epsilon }}_{X,v}-{\tilde{\epsilon }}_{X}\right]\;\\ \;\left[\alpha_{Y,v}+\eta_{Y,v}{\bar{\omega }}_{Y}+{\bar{\epsilon }}_{Y,v}-{\tilde{\epsilon }}_{Y}\right] \end{array}}{\begin{array}{l} \sqrt{\sum_{v=1}^{V}{\left[\alpha_{X,v}+\eta_{X,v}{\bar{\omega }}_{X}+{\bar{\epsilon }}_{X,v}-{\tilde{\epsilon }}_{X}\right]}^{2}}\\ \;\sqrt{\sum_{v=1}^{V}{\left[\alpha_{Y,v}+\eta_{Y,v}{\bar{\omega }}_{Y}+{\bar{\epsilon }}_{Y,v}-{\tilde{\epsilon }}_{Y}\right]}^{2}} \end{array}},$$ (5)

where ${\tilde{\epsilon }}_{X}\;=\sum_{v=1}^{V}{\bar{\epsilon }}_{X,v}/V$ and ${\tilde{\epsilon }}_{Y}=\sum_{v=1}^{V}{\bar{\epsilon }}_{Y,v}/V$ . The subscript V in ${\text{Corr}}_{V}\left(\cdot ,\cdot \right)$ emphasizes that the correlation is computed across all brain locations V={1, …, V} under study. Because the correlation is calculated across spatial locations, any terms that are constant with respect to *v*, including *μ_X_*, *μ_Y_*, ${\bar{\beta }}_{X}$, and ${\bar{\beta }}_{Y}$, are canceled out by spatial centering and, therefore, do not contribute to the correlation.

Because the random effects, $\beta_{X}^{s}$, $\beta_{Y}^{s}$, $\omega_{X}^{s}$, and $\omega_{Y}^{s}$, and residuals, $\epsilon_{X,v}^{s}$ and $\epsilon_{Y,v}^{s}$, are independent across subjects and have zero means, the law of large numbers implies that for each location *v*,

$$\begin{array}{l} {\bar{\beta }}_{X},\;{\bar{\beta }}_{Y},\;{\bar{\omega }}_{X},\;{\bar{\omega }}_{Y},\;{\bar{\epsilon }}_{X,v},\;{\bar{\epsilon }}_{Y,v}\;\overset{S\to \infty }{\to }E\left(\beta_{X}^{s}\right)=E\left(\beta_{Y}^{s}\right)=E\left(\omega_{X}^{s}\right)\\ \;=E\left(\omega_{Y}^{s}\right)=E\left(\epsilon_{X,v}^{s}\right)=E\left(\epsilon_{Y,v}^{s}\right)=0. \end{array}$$ (6)

Thus, in studies with a large sample size *S* (more than a hundred), these terms become negligible, and the correlation between the group-average brain maps,

$$\hat{\rho }={\text{Corr}}_{V}\left({\bar{X}}_{v},\;{\bar{Y}}_{v}\right),$$

is a close estimate of the correlation between the population-mean *X* and *Y* brain maps:

$$\begin{array}{l} {\text{ρ = Corr}}_{\nu }\left(\mu_{X}+\beta_{X,v},\mu_{Y}+\alpha_{Y,v}\right)\\ {\text{ = Corr}}_{\nu }\left(\alpha_{X,v},\;\alpha_{Y,v}\right)=\frac{\sum_{v=1}^{V}\alpha_{X,v}\;\alpha_{Y,v}}{\sqrt{\sum_{v=1}^{V}\alpha_{X,v}^{2}}\sqrt{\sum_{v=1}^{V}\alpha_{Y,v}^{2}}}. \end{array}$$

Importantly, this definition of ρ depends only on the assumption that the population-mean brain maps are fixed across subjects and does not rely on the specific form of the mixed-effects models (1)–(2).

#### 2.5.2 The spin test

The spin test (Alexander-Bloch et al., 2018) assesses the significance of the observed correlation $\hat{\rho }={\text{Corr}}_{V}\left({\bar{X}}_{v},\;{\bar{Y}}_{v}\right)$ by comparing it against an empirical null distribution constructed from spatially rotated surrogate maps. Each surrogate is obtained by applying a random rotation on the cortical surface to one of the two maps (say *X*), while holding the other map *Y* fixed.

Let π:V→V denote the permutation of spatial-location indices induced by such a rotation, so that *π*(*v*) is the index mapped to location *v*. Under model (1), the rotated group-average map is

$${\bar{X}}_{\pi \left(v\right)}=\mu_{X}+\alpha_{X,\pi \left(v\right)}+{\bar{\beta }}_{X}+\eta_{X,\pi \left(v\right)}{\bar{\omega }}_{X}+{\bar{\epsilon }}_{X,\pi \left(v\right)},$$

and the correlation between this rotated *X* map and the original *Y* map is

$$Corr_{V}\left({\bar{X}}_{\pi \left(v\right)},\;{\bar{Y}}_{v}\right)=\frac{{\text{Cov}}_{V}\left(\alpha_{X,\pi \left(v\right)}+\eta_{X,\pi \left(v\right)}{\bar{\omega }}_{X}+{\bar{\epsilon }}_{X,\pi \left(v\right)},\alpha_{Y,v}+\eta_{Y,v}{\bar{\omega }}_{Y}+{\bar{\epsilon }}_{Y,v}\right)}{\sqrt{Var_{V}\left(\alpha_{X,v}+\eta_{X,v}{\bar{\omega }}_{X}+{\bar{\epsilon }}_{X,v}\right)}\sqrt{Var_{V}\left(\alpha_{Y,v}+\eta_{Y,v}{\bar{\omega }}_{Y}+{\bar{\epsilon }}_{Y,v}\right)}}.$$ (7)

Because *π* merely reorders the *V* locations, it preserves the empirical variance of each map; the denominator of (7), therefore, coincides with that of (5).

Collecting this statistic over all rotations yields the empirical null distribution

$$\hat{S}=\left\{{\text{Corr}}_{V}\left({\bar{X}}_{\pi \left(v\right)},{\bar{Y}}_{v}\right),\;\text{for all}\;\pi \right\}.$$

Let ${\hat{L}}_{a/2}$ and Û*_a_*_/2_ be its lower and upper *a*/2 percentiles. The spin test rejects when

$$\hat{\rho }\;<{\hat{L}}_{a/2}\text{ or }\hat{\rho }>\;{\hat{U}}_{a/2}.$$ (8)

As the number of subjects *S* grows, the law of large numbers (Eq. (6)) drives the subject-averaged terms ${\bar{\omega }}_{X}$ and ${\bar{\epsilon }}_{X,\pi \left(v\right)}$ to zero. Consequently,

$$\begin{array}{l} {\text{Corr}}_{V}\left({\bar{X}}_{\pi \left(v\right)},\;{\bar{Y}}_{v}\right)\;\overset{S\to \infty }{\to }{\text{Corr}}_{V}\left(\mu_{X}+\alpha_{X,\pi \left(v\right)},\;\mu_{Y}+\alpha_{Y,v}\right)\\ \;=\frac{\sum_{v=1}^{V}\alpha_{X,\pi \left(v\right)}\alpha_{Y,v}}{\sqrt{\sum_{v=1}^{V}\alpha_{X,v}^{2}}\sqrt{\sum_{v=1}^{V}\alpha_{Y,v}^{2}}}. \end{array}$$

Thus, for large *S*, the correlation between rotated group-average maps is well approximated by the correlation between the rotated population-mean maps, which depends only on the spatial effects α_X, *v*_ and α_Y, *v*_.

For mathematical simplicity and clarity, we work throughout in this large-*S* regime (with *S* at least in the hundreds), where the law of large numbers gives

$$\hat{\rho }\approx \rho ,\;\;\;\;\;\;\hat{S}\approx S,{\hat{L}}_{a/2}\;\approx L_{a/2},\;\;{\hat{U}}_{a/2}\;\approx U_{a/2},$$

with $S=\left\{{\text{Corr}}_{V}\left(\alpha_{X,\pi \left(v\right)},\;\alpha_{Y,v}\right),\text{ for all }\pi \right\}$ and *L_a_*_/2_, *U_a_*_/2_ its lower and upper *a*/2 percentiles. Hence, for large *S*, 𝔖 is the null distribution of the spin test, and the test statistic $\hat{\rho }$ is essentially a constant.

The spin test treats $\hat{\rho }$ as a draw from $\hat{S}$ under the null hypothesis. In the large-*S* regime, however, $\hat{\rho }\to \rho$ is a constant, so it can be distributed according to 𝔖 only if 𝔖 is degenerate at ρ. Equivalently, the test statistic follows 𝔖 if and only if

$${\text{Corr}}_{V}\left(\alpha_{X,\pi \left(v\right)},\;\alpha_{Y,v}\right)=\rho \;\text{for all }\pi .$$ (9)

Averaged over rotations, we have $E_{\pi }\left(\alpha_{X,\pi \left(v\right)}\right)={\bar{\alpha }}_{X}=0$ , since the spatial effects are centered ($\sum_{v=1}^{V}\alpha_{X,v}=0$ ). Therefore,

$$E_{\pi }\left({\text{Corr}}_{V}\left(\alpha_{X,\pi \left(v\right)},\;\alpha_{Y,v}\right)\right)=\frac{\sum_{v=1}^{V}E_{\pi }\left(\alpha_{X,\pi \left(v\right)}\right)\alpha_{Y,v}}{\sqrt{\sum_{v=1}^{V}\alpha_{X,v}^{2}}\sqrt{\sum_{v=1}^{V}\alpha_{Y,v}^{2}}}=0.$$

Taking the expectation over *π* on both sides of (9) gives ρ = 0, so condition (9) is equivalent to

$${\text{Corr}}_{V}\left(\alpha_{X,\pi \left(v\right)},\;\alpha_{Y,v}\right)=0\;\text{for all }\pi ,$$

and the associated null hypothesis of the spin test is given by

$$H_{0}:{\text{Corr}}_{V}\left(\alpha_{X,v},\;\alpha_{Y,v}\right)=0.$$ (10)

For 𝔖 to serve as the null distribution of the test statistic in the large-*S* regime, condition (9) has to hold under *every* rotation, and each rotated configuration $\left\{\alpha_{X,\pi \left(1\right)},\;\ldots ,\;\alpha_{\text{X},\pi \left(V\right)}\right\}$ must be statistically equivalent to the original, that is, a realization of the same spatial process. This equivalence holds only when $\left\{\alpha_{X,v},\;v=1,\;\ldots ,\;V\right\}$ is a realization of a spatially stationary process, which means that its autocorrelation structure is homogeneous across spatial locations.

Even under stationarity, the empirical correlation ${\text{Corr}}_{V}\left(\alpha_{X,\pi \left(v\right)},\;\alpha_{Y,v}\right)$ varies from realizations, that is, from rotations. Its variance across rotations decreases with the number of observations from each map, *V*, so these values concentrate at zero only as *V* → ∞. In this idealized regime, the null distribution 𝔖 collapses to a point mass at 0, and the spin test can be rejected for every nonzero ρ.

In practice, however, population-mean brain maps are spatially *nonstationary*, and the number of locations *V* is *finite*. Under these conditions ${\text{Corr}}_{V}\left(\alpha_{X,\pi \left(v\right)},\;\alpha_{Y,v}\right)$ varies substantially across rotations and takes nonzero values, so 𝔖 remains dispersed around zero rather than degenerating. The acceptance region $\left[L_{a/2},\;U_{a/2}\right]$ is then a nontrivial interval, and the spin test fails to reject (10) whenever

$$\rho \in \left[L_{a/2},\;U_{a/2}\right].$$ (11)

As a result, even a genuinely nonzero correlation ρ can go undetected, making the spin test conservative precisely when stationarity fails or *V* is small.

#### 2.5.3 BrainSMASH

BrainSMASH (Burt et al., 2020) assesses correspondence between population-mean brain maps using the same test statistic as the spin test, $\hat{\rho }={\text{Corr}}_{V}\left({\bar{X}}_{v},\;{\bar{Y}}_{v}\right)$, but differs fundamentally in its null model construction. Instead of rotating maps on the cortical surface, BrainSMASH generates surrogate maps that preserve the overall spatial autocorrelation of the target map ${\bar{X}}_{v}$.

The procedure begins by randomly permuting the values of ${\bar{X}}_{v}$ across all locations, followed by spatial smoothing and rescaling to reproduce the autocorrelation of the original map. This produces surrogate maps that retain similar spatial smoothness of ${\bar{X}}_{v}$ but disrupt spatial alignment with *Yˉ_v_*.

Let ${\overset{⌣}{X}}_{v}$ denote the value at location *v* in one surrogate *X* brain map. BrainSMASH constructs ${\overset{⌣}{X}}_{v}$ as

$${\overset{⌣}{X}}_{v}\;=\sum_{j=1}^{V}w_{v,P\left(j\right)}{\bar{X}}_{j}+w_{0}z_{v},$$ (12)

where (P(1), …, P(V)) is a random permutation of (1, …, V), *P*(*j*) denotes the new location index of the brain map value at the original location *j* after the permutation, *w_v_*_, *P*(*j*)_ is the spatial smoothing and rescaling coefficient for ${\bar{X}}_{j}$ in the surrogate map at location *v*, *z_v_*, v=1, …, V , are i.i.d. with *N* (0, 1), and *w*_0_ is its scaling coefficient.

Let

$$\hat{B}=\left\{{\text{Corr}}_{V}\left({\overset{⌣}{X}}_{v},{\bar{Y}}_{v}\right)\text{ for all permutations }P\right\}$$

denote the set of all permutation-based statistics, that is, the empirical null distribution generated by BrainSMASH. Let ${\hat{L}}_{a/2}^{B}$ and ${\hat{U}}_{a/2}^{B}$ denote the lower and upper *a*/2 percentiles of $\hat{B}$ . BrainSMASH rejects if

$$\hat{\rho }\;<{\hat{L}}_{a/2}^{B}\text{ or }\hat{\rho }\;>{\hat{U}}_{a/2}^{B}.$$ (13)

Under the mixed-effects model (1), substituting the expression for ${\bar{X}}_{j}$ in (12) yields

$${\overset{⌣}{X}}_{v}\;=\left(\mu_{X}\;+{\bar{\beta }}_{X}\right)c_{X}+\sum_{j=1}^{V}w_{v,P\left(j\right)}\left(\alpha_{X,j}\;+\eta_{X,j}{\bar{\omega }}_{X}\;+{\bar{\epsilon }}_{X,j}\right)+w_{0}z_{v},$$

where $c_{X}=\sum_{j=1}^{V}w_{v,P\left(j\right)}$ is a constant that depends only on the smoothing kernel.

When the number of subjects *S* is large (in hundreds), the law of large numbers (Eq. (6)) ensures that ${\bar{\beta }}_{X}$, ${\bar{\omega }}_{X}$, and ${\bar{\epsilon }}_{X,j}$ are close to zero, and ${\overset{⌣}{X}}_{v}$ roughly equals the permuted population-mean brain map:

$${\overset{⌣}{X}}_{v}\approx \mu_{X}c_{X}+\sum_{j=1}^{V}w_{v,P\left(j\right)}\;\alpha_{X,j}+w_{0}z_{v}.$$

In addition, the correlation between ${\overset{⌣}{X}}_{v}$ and Yˉ*_v_*, ${\text{Corr}}_{V}\left({\overset{⌣}{X}}_{v},\;{\bar{Y}}_{v}\right)$, closely approximates

$$\begin{array}{l} {\text{Corr}}_{V}(\sum_{j=1}^{V}w_{v,P\left(j\right)}\alpha_{X,j}+w_{0}z_{v},\alpha_{Y,v})\\ \;=\frac{{\text{Cov}}_{V}\left(\sum_{j=1}^{V}w_{v,P\left(j\right)}\alpha_{X,j}\;+w_{0}z_{v},\alpha_{Y,v}\right)}{\sqrt{{\text{Var}}_{V}(\sum_{j=1}^{V}w_{v,P\left(j\right)}\alpha_{X,j}\;+w_{0}z_{v})}\sqrt{{\text{Var}}_{V}\left(\alpha_{Y,v}\right)}}. \end{array}$$ (14)

Consequently, for a sufficiently large *S*,

$$\begin{array}{l} B=\{{\text{Corr}}_{V}(\sum_{j=1}^{V}w_{v,P\left(j\right)}\alpha_{X,j}+w_{0}\;z_{v},\alpha_{Y,v})\;\\ \;\;\;\text{for all permutations }P\} \end{array}$$

forms the null distribution for BrainSMASH, which is rejected when

$$\rho \;<L_{a/2}^{B}\text{or }\rho >\;U_{a/2}^{B},$$

where $L_{a/2}^{B}$ and $U_{a/2}^{B}$ denote the lower and upper *a*/2 percentiles of 𝔅, respectively.

##### The implicit null hypothesis

The argument now proceeds exactly as for the spin test. In the large-*S* regime, $\hat{\rho }\to \rho$ has vanishing variance, and a degenerate random variable can follow 𝔅 only if 𝔅 is itself degenerate. Hence the test statistic follows 𝔅 if and only if

$${\text{Corr}}_{V}(\sum_{j=1}^{V}w_{v,P\left(j\right)}\alpha_{X,j}+w_{0}z_{v},\alpha_{Y,v})=\rho \;\text{for all permutations}\;P.$$ (15)

The constant is pinned at zero by the same centering argument used for the spin test. Because the spatial effects are centered, $\sum_{j=1}^{V}\alpha_{X,j}=0$ , and $E_{P}\left(w_{v,P\left(j\right)}\right)={\bar{w}}_{v}$ does not depend on *j*, we have

$$E_{P}\left(\sum_{j=1}^{V}w_{v,P\left(j\right)}\alpha_{X,j}+w_{0}z_{v}\right)={\bar{w}}_{v}\sum_{j=1}^{V}\alpha_{X,j}=0.$$

Therefore, BrainSMASH tests the same null hypothesis as the spin test,

$$H_{0}:{\text{Corr}}_{V}\left(\alpha_{X,v},\;\alpha_{Y,v}\right)=0.$$ (16)

If the population-mean brain maps are spatially stationary and the number of observations for each map, that is, the number of spatial locations *V*, is sufficiently large, the correlations in 𝔅 have little variation and concentrate around their expectation, namely zero.

In practice, however, population-mean brain maps are generally nonstationary, and the effective spatial sample size may not be sufficiently large. Consequently, the correlations

$${\text{Corr}}_{V}\left(\sum_{j=1}^{V}w_{v,P\left(j\right)}\alpha_{X,j}\;+w_{0}z_{v},\alpha_{Y,v}\right)$$

can vary substantially across surrogate maps, producing a diffuse permutation null distribution with

$$L_{a/2}^{B}<0<U_{a/2}^{B}.$$

As a result, moderately nonzero population correlations may still lie within the empirical non-rejection region

$$\rho \in \left[L_{a/2}^{B},\;U_{a/2}^{B}\right],$$

leading to conservative inference and reduced statistical power.

#### 2.5.4 The SPICE test

The SPICE test (Weinstein et al., 2021) differs fundamentally from the spin test and BrainSMASH in both its test statistic and its permutation scheme. Rather than comparing group-average brain maps, the SPICE test assesses correspondence at the individual-subject level. Specifically, it uses the average correlation between each subject’s *X* and *Y* brain maps as the test statistic and compares it with a null distribution generated by randomly permuting subject pairings across *X* and *Y* brain maps.

Let *k* denote a permutation of subject indices {1, …, S}, and let *k_s_* denote the subject index paired with subject *s* after permutation. We derive the permutation statistic *A_k_* and the test statistic *A*_0_ of the SPICE test under the mixed-effects models (1) and (2) below.

The correlation between the *X* brain map of subject *k_s_* and the *Y* brain map of subject *s* is given by

$${\text{Corr}}_{V}\left(X_{v}^{k_{s}},\;Y_{v}^{s}\right)=\frac{{\text{Cov}}_{V}\left(X_{v}^{k_{s}},\;Y_{v}^{s}\right)}{\sqrt{T_{X}^{k_{s}}T_{Y}^{s}}},$$

where

$$\begin{array}{l} T_{X}^{k_{s}}={\text{Var}}_{V}\left(\alpha_{X,v}\;+\eta_{X,v}\omega_{X}^{k_{s}}\;+\epsilon_{X,v}^{k_{s}}\right),\;\;\\ T_{Y}^{s}={\text{Var}}_{V}\left(\alpha_{Y,v}\;+\eta_{Y,v}\omega_{Y}^{s}\;+\epsilon_{Y,v}^{s}\right). \end{array}$$

The covariance ${\text{Cov}}_{V}\left(X_{v}^{k_{s}},\;Y_{v}^{s}\right)$ in the numerator can be expanded under the mixed-effects model as

$$\begin{array}{l} {\text{Cov}}_{V}\left(\alpha_{X,v},\;\alpha_{Y,v}\right)+\omega_{X}^{k_{s}}\omega_{Y}^{s}{\text{ Cov}}_{V}\left(\eta_{X,v},\;\eta_{Y,v}\right)\\ \;+{\text{Cov}}_{V}\left(\alpha_{X,v},\;\eta_{Y,v}\right)\omega_{Y}^{s}+\omega_{X}^{k_{s}}{\text{Cov}}_{V}\left(\eta_{X,v},\;\alpha_{Y,v}\right)+E^{k_{s},s}, \end{array}$$

where the remainder term $E^{k_{s},s}$ captures the covariances involving residuals:

$$\begin{array}{l} E^{k_{s},s}\;={\text{Cov}}_{V}\left(\epsilon_{X,v}^{k_{s}},\;\alpha_{Y,v}\right)+\omega_{Y}^{s}{\text{Cov}}_{V}\left(\epsilon_{X,v}^{k_{s}},\;\eta_{Y,v}\right)\\ \;\;\;\;\;+{\text{Cov}}_{V}\left(\alpha_{X,v},\;\epsilon_{Y,v}^{s}\right)+\omega_{X}^{k_{s}}{\text{Cov}}_{V}\left(\eta_{X,v},\;\epsilon_{Y,v}^{s}\right)\\ \;\;\;\;\;+{\text{Cov}}_{V}\left(\epsilon_{X,v}^{k_{s}},\;\epsilon_{Y,v}^{s}\right). \end{array}$$

Averaging these correlations across all paired subjects yields the SPICE permutation statistic (*A_k_*) and test statistic (*A*_0_):

$$A_{k}=\frac{1}{S}\sum_{s=1}^{S}{\text{Corr}}_{V}\left(X_{v}^{k_{s}},Y_{v}^{s}\right)\;\;\text{and}\;A_{0}=\frac{1}{S}\sum_{s=1}^{S}{\text{Corr}}_{V}\left(X_{v}^{s},Y_{v}^{s}\right).$$

Here, *A*_0_ is the average correlation between each subject’s own pair of *X* and *Y* maps, while *A_k_* represents the average under random cross-subject pairings generated by permutation *k*.

As the number of subjects *S* increases, by the law of large numbers and the independence of random effects and residuals across subjects, both statistics converge to their expected values:

$$A_{k}\overset{S\to \infty }{\to }E\left[{\text{Corr}}_{V}\left(X_{v}^{k_{s}},Y_{v}^{s}\right)]\;\;\text{and}\;\;A_{0}\overset{S\to \infty }{\to }E[{\text{Corr}}_{V}\left(X_{v}^{s},Y_{v}^{s}\right)\right].$$ (17)

The two expectations in Eq. (17) become identical when ${\text{Corr}}_{V}\left(X_{v}^{k_{s}},\;Y_{v}^{s}\right)$ and ${\text{Corr}}_{V}\left(X_{v}^{s},\;Y_{v}^{s}\right)$ follow the same distribution. Since random effects and residuals from different subjects are independent:

$$\begin{array}{l} \left(\omega_{X}^{k_{s}},\in_{X,1}^{k_{s}},\cdots ,\in_{X,V}^{k_{s}}\right)⊥\left(\omega_{Y}^{s},\in_{Y,1}^{s},\cdots ,\in_{Y,V}^{s}\right),\\ X_{v}^{k_{s}}⊥Y_{v}^{s},{\text{ and T}}_{X}^{k_{s}}⊥T_{Y}^{s}, \end{array}$$

this equivalence holds when all subject-specific location-related random variables in models (1) and (2) are independent. Consequently, the null hypothesis of the SPICE test is given by

$$H_{0}:\eta_{X,v}\omega_{X}^{s}+\epsilon_{X,v}^{s}\;⊥\;\eta_{Y,v^{\prime }}\;\omega_{Y}^{s}+\epsilon_{Y,v^{\prime }}^{s}\text{ for all }v,\;\text{and}\;v^{\prime }.$$ (18)

This formulation indicates that the SPICE test specifically evaluates independence between subject-specific spatial variability in two brain maps.

### 2.6 Implementation of permutation tests

We used the default settings of publicly available packages to implement the spin test, BrainSMASH, and the SPICE test. All analyses were performed separately for the left and right hemispheres, because these tests operate on hemisphere-specific spherical projections of the cortical surface.

#### 2.6.1 The Spin test

We used the publicly available toolbox by Alexander-Bloch et al. (2018) (https://github.com/spin-test/spin-test) to generate vertex-wise rotations and used the adaptation of the method by Váša et al. (2018) (https://github.com/frantisekvasa/rotate_parcellation) to generate parcel-level rotations. For each spin test, 1,000 rotations of the data were performed.

#### 2.6.2 BrainSMASH

We used the BrainSMASH Python package (Burt et al., 2020) (https://github.com/murraylab/brainsmash) to generate 1,000 surrogate maps and implement BrainSMASH. We followed the recommended workflows for analyzing parcel-level and vertex-wise data and verified surrogate fidelity via the variogram envelope check (Supplementary Fig. S2).

#### 2.6.3 The SPICE test

We generated the null distribution of intermodal correspondence statistics by randomly permuting the subject indices of the first modality 1,000 times (Phipson & Smyth, 2010). The SPICE test was implemented using the authors’ open-source software (Weinstein et al., 2021) (https://github.com/smweinst/spice_test).

### 2.7 Bootstrap-based inference procedures

Instead of constructing null distributions for test statistics, as done in the permutation tests, we adopt a nonparametric bootstrap approach (Efron, 1992) to directly assess the sampling variability of estimates of correspondence between brain maps. The bootstrap-based methods construct confidence intervals for correspondence measures, which in turn allows formal hypothesis testing. Specifically, if these confidence intervals exclude the zero value that represents no correspondence, the null hypothesis is rejected.

In the following, we describe the bootstrap-based method for constructing confidence intervals and performing hypothesis testing to make inferences about correspondence between population-mean brain maps and between subject-specific brain maps.

#### 2.7.1 Evaluating correspondence between population-mean brain maps

We use the statistic

$$\hat{\rho }={\text{Corr}}_{V}\left({\bar{X}}_{v},\;{\bar{Y}}_{v}\right)$$

as the estimator of the correlation between population-mean brain maps $\rho ={\text{Corr}}_{V}\left(\alpha_{X,v},\alpha_{Y,v}\right)$, which measures the correspondence between population-mean brain maps.

To assess the variability of $\hat{\rho }$ , we perform 500 independent bootstrap resamples. In each resample, *S* subjects’ brain maps are drawn with replacement from the original dataset. For the *b*th bootstrap iteration, denote the resampled data as $X_{v}^{s\left(b\right)}$ and $Y_{v}^{s\left(b\right)}$, for v=1, …, V and s=1, …,S . We compute group-average brain maps and their correlation as

$$\begin{array}{l} {\bar{X}}_{v}^{\left(b\right)}=\sum_{s=1}^{S}X_{v}^{s\left(b\right)}/S\;\text{and }{\bar{Y}}_{v}^{\left(b\right)}=\sum_{s=1}^{S}Y_{v}^{s\left(b\right)}/S\;\text{and }\\ {\hat{\rho }}^{\left(b\right)}={\text{Corr}}_{V}\left({\bar{X}}_{v}^{\left(b\right)},\;{\bar{Y}}_{v}^{\left(b\right)}\right). \end{array}$$

The empirical distribution of $\left\{{\hat{\rho }}^{\left(b\right)},\;b=1,\;\ldots ,\;500\right\}$ is then used to construct a 95% confidence interval for ρ.

If this interval does not cover zero, we reject the null hypothesis

$$H_{0}:{\text{Corr}}_{V}\left(\alpha_{X,v},\;\alpha_{Y,v}\right)=0.$$ (19)

#### 2.7.2 Evaluating correspondence between subject-specific brain maps

Under models (1) and (2), correspondence between subject-specific brain maps arises in different forms, which are characterized by various correlations between different subject-specific parameters, including the correlation between main subject effects $\beta_{X}^{s}$ and $\beta_{Y}^{s}$, between subject-specific variability in the dominant spatial patterns, that is, interaction subject effects $\omega_{X}^{s}$ and $\omega_{Y}^{s}$, between residuals $\epsilon_{X,v}^{s}$ and $\epsilon_{Y,v}^{s}$, and also between interaction subject effects and residuals. These correlations are defined below:

$$\begin{array}{l} \rho_{\beta ,\beta }=\text{Corr}\left(\beta_{X}^{s},\;\beta_{Y}^{s}\right)=\underset{S\to \infty }{\text{lim}}{\text{Corr}}_{S}\left(\beta_{X}^{s},\;\beta_{Y}^{s}\right),\\ \rho_{\omega ,\omega }=\text{Corr}\left(\omega_{X}^{s},\;\omega_{Y}^{s}\right)=\underset{S\to \infty }{\text{lim}}{\text{Corr}}_{S}\left(\omega_{X}^{s},\;\omega_{Y}^{s}\right),\\ \rho_{\epsilon ,\epsilon }=\frac{1}{V}\sum_{v=1}^{V}\text{Corr}\left(\epsilon_{X,v}^{s},\;\epsilon_{Y,v}^{s}\right)=\underset{S\to \infty }{\text{lim}}\frac{1}{V}\sum_{v=1}^{V}{\text{Corr}}_{S}\left(\epsilon_{X,v}^{s},\;\epsilon_{Y,v}^{s}\right),\\ \rho_{\epsilon ,\omega }=\frac{1}{V}\sum_{v=1}^{V}\text{Corr}\left(\epsilon_{X,v}^{s},\;\omega_{Y}^{s}\right)=\underset{S\to \infty }{\text{lim}}\frac{1}{V}\sum_{v=1}^{V}{\text{Corr}}_{S}\left(\epsilon_{X,v}^{s},\;\omega_{Y}^{s}\right),\\ \rho_{\omega ,\epsilon }=\frac{1}{V}\sum_{v=1}^{V}\text{Corr}\left(\omega_{X}^{s},\;\epsilon_{Y,v}^{s}\right)=\underset{S\to \infty }{\text{lim}}\frac{1}{V}\sum_{v=1}^{V}{\text{Corr}}_{S}\left(\omega_{X}^{s},\;\epsilon_{Y,v}^{s}\right), \end{array}$$

where the subscript S in ${\text{Corr}}_{S}\left(\cdot ,\cdot \right)$ indicates that the correlation is calculated across the *S* subjects in the data, defined as

$${\text{Corr}}_{S}\left(\theta_{X}^{s},\;\phi_{Y}^{s}\right)=\frac{\sum_{s=1}^{S}\left(\theta_{X}^{s}-{\bar{\theta }}_{X}\right)\left(\phi_{Y}^{s}-{\bar{\phi }}_{Y}\right)}{\sqrt{\sum_{s=1}^{S}{\left(\theta_{X}^{s}-{\bar{\theta }}_{X}\right)}^{2}}\sqrt{\sum_{s=1}^{S}{\left(\phi_{Y}^{s}-{\bar{\phi }}_{Y}\right)}^{2}}},$$

and Corr(·,·) denotes the correlation calculated across all subjects in the population and is the limit of ${\text{Corr}}_{S}\left(\cdot ,\cdot \right)$:$\text{Corr}\left(\cdot ,\cdot \right)=\underset{S\to \infty }{\text{lim}}{\text{Corr}}_{S}\left(\theta_{X}^{s},\phi_{Y}^{s}\right)$.

To estimate the correlations described above, we first estimate all effects in Eqs. (1)–(2) by minimizing the sum of squared errors with respect to the model parameters. For each modality, after removing the estimated global mean, main spatial effects, and main subject effects, we form the *S* × *V* matrix of remaining subject-by-location variation. The interaction term, $\eta_{X,v}\omega_{X}^{s}$ or $\eta_{Y,v}\omega_{Y}^{s}$, defined in Section 2.4 as the dominant subject-by-location interaction, is estimated as the best rank-one approximation to this matrix. By the Eckart–Young–Mirsky theorem, the leading component of the singular value decomposition gives this least-squares solution. The estimated interaction subject effects are standardized to have empirical variance one, with the corresponding scale absorbed into the interaction spatial effects. Variation not captured by this interaction component is included in the residual term. This estimation procedure yields the following estimates:

$${\hat{\mu }}_{X},\;{\hat{\alpha }}_{X,v},\;{\hat{\beta }}_{X}^{s},\;{\hat{\eta }}_{X,v},\;{\hat{\omega }}_{X}^{s},\;{\hat{\mu }}_{Y},\;{\hat{\alpha }}_{Y,v},\;{\hat{\beta }}_{Y}^{s},\;{\hat{\eta }}_{Y,v},\;{\hat{\omega }}_{Y}^{s}.$$

The corresponding estimates of model residuals are

$$\begin{array}{c} {\hat{\epsilon }}_{X,v}^{s}=X_{v}^{s}-{\hat{\mu }}_{X}-{\hat{\alpha }}_{X,v}-{\hat{\beta }}_{X}^{s}-{\hat{\eta }}_{X,v}{\hat{\omega }}_{X}^{s},\;\\ {\hat{\epsilon }}_{Y,v}^{s}=Y_{v}^{s}-{\hat{\mu }}_{Y}-{\hat{\alpha }}_{Y,v}-{\hat{\beta }}_{Y}^{s}-{\hat{\eta }}_{Y,v}{\hat{\omega }}_{Y}^{s}. \end{array}$$

Using these values, we estimate ρ_β,β_, ρ_ω,ω_, ρ_ϵ,ϵ_, ρ_ϵ,ω_, ρ_ω,ϵ_ by

$$\begin{array}{llll} \begin{array}{l} {\hat{\rho }}_{\beta ,\beta }={\text{Corr}}_{S}\left({\hat{\beta }}_{X}^{s},\;{\hat{\beta }}_{Y}^{s}\right),\;{\hat{\rho }}_{\omega ,\omega }={\text{Corr}}_{S}\left({\hat{\omega }}_{X}^{s},\;{\hat{\omega }}_{Y}^{s}\right),\;\\ {\hat{\rho }}_{\epsilon ,\epsilon }=\sum_{v=1}^{V}{\text{Corr}}_{S}\left({\hat{\epsilon }}_{X,v}^{s},\;{\hat{\epsilon }}_{Y,v}^{s}\right)/V, \end{array} & & & \\ \begin{array}{l} {\hat{\rho }}_{\epsilon ,\omega }=\sum_{v=1}^{V}{\text{Corr}}_{S}\left({\hat{\epsilon }}_{X,v}^{s},\;{\hat{\omega }}_{Y}^{s}\right)/V,\;\;\text{and }\\ {\hat{\rho }}_{\omega ,\epsilon }=\sum_{v=1}^{V}{\text{Corr}}_{S}\left({\hat{\omega }}_{X}^{s},\;{\hat{\epsilon }}_{Y,v}^{s}\right)/V. \end{array} & & & \end{array}$$

We apply the same estimation procedure to bootstrap samples $X_{v}^{s\left(b\right)}$ and $Y_{v}^{s\left(b\right)}$ for v=1, …,V and s=1, …,S , obtaining bootstrap-sample-based estimates

$${\hat{\rho }}_{\beta ,\beta }^{\left(b\right)},\;{\hat{\rho }}_{\omega ,\omega }^{\left(b\right)},\;{\hat{\rho }}_{\epsilon ,\epsilon }^{\left(b\right)},\;{\hat{\rho }}_{\epsilon ,\omega }^{\left(b\right)},\;\text{and }{\hat{\rho }}_{\omega ,\epsilon }^{\left(b\right)}$$

for b=1, …, 500 .

Based on the bootstrap estimates $\left\{{\hat{\rho }}_{\beta ,\beta }^{\left(b\right)},\;b=1,\;\ldots ,\;500\right\}$, $\left\{{\hat{\rho }}_{\omega ,\omega }^{\left(b\right)},\;b=1,\;\ldots ,\;500\right\}$, $\left\{{\hat{\rho }}_{\epsilon ,\epsilon }^{\left(b\right)},\;b=1,\;\ldots ,\;500\right\}$, $\left\{{\hat{\rho }}_{\in ,\omega }^{\left(b\right)},b=1,\ldots ,500\right\}$ , $\left\{{\hat{\rho }}_{\omega ,\epsilon }^{\left(b\right)},\;b=1,\;\ldots ,\;500\right\}$, we construct 95% confidence intervals for the corresponding correlations: ρ_β,β_, ρ_ω,ω_, ρ_ϵ,ϵ_, ρ_ϵ,ω_, and ρ_ω,ϵ_, respectively.

To test the null hypothesis of no correspondence between subject-specific brain maps, we consider several tests of independence between subject-specific parameters:

$$\begin{array}{l} H_{0}^{1}:\;\beta_{X}^{s}\;⊥\;\beta_{Y}^{s},\;H_{0}^{2}:\omega_{X}^{s}⊥\omega_{Y}^{s},\;H_{0}^{3}:\epsilon_{X,v}^{s}⊥\epsilon_{Y,v}^{s},\;\\ H_{0}^{4}:\epsilon_{X,v}^{s}⊥\omega_{Y}^{s},\;\text{and}\;H_{0}^{5}:\omega_{X}^{s}⊥\epsilon_{Y,v}^{s}. \end{array}$$

We test $H_{0}^{1}$ based on the confidence interval for ρ_β,β_, test $H_{0}^{2}$ based on the confidence interval for ρ_ω,ω_, test $H_{0}^{3}$ based on the confidence interval for ρ_ϵ,ϵ_, test $H_{0}^{4}$ based on the confidence interval for ρ_ϵ,_*_ω_*, and test $H_{0}^{5}$ based on the confidence interval for ρ_ω,ϵ_. Specifically, each null hypothesis of independence is rejected if the 95% confidence interval for the associated correlation parameter does not include zero. To evaluate the overall hypothesis of no correspondence between subject-specific brain maps, we apply a Bonferroni correction across all confidence intervals to control the family-wise error rate. We refer to this procedure with Bonferroni correction as the *overall* bootstrap-based (inference) method.

Note that the null hypothesis of the SPICE test (18) is mathematically equivalent to simultaneously testing $H_{0}^{2}$ to $H_{0}^{5}$.

## 3 Simulation Studies

We conducted simulation studies to evaluate the type I error rate and statistical power of the proposed bootstrap-based inference method and to compare its performance with three widely used permutation-based methods: the spin test, BrainSMASH, and the SPICE test. All methods were evaluated at the 5% significance level.

We first compared the bootstrap-based method with the spin test and BrainSMASH for testing correspondence between population-mean brain maps under the null hypothesis

$$H_{0}:{\text{Corr}}_{V}\left(\alpha_{X,v},\;\alpha_{Y,v}\right)=0.$$

We then compared the bootstrap-based method with the SPICE test for assessing correspondence between subject-specific brain maps under the following three null hypotheses:

$$H_{0}^{1}\;:\;\beta_{X}^{s}⊥\beta_{Y}^{s},\;\;\;\;\;H_{0}^{2}\;:\omega_{X}^{s}⊥\omega_{Y}^{s},\;\;H_{0}^{3}\;:\epsilon_{X,v}^{s}⊥\epsilon_{Y,v}^{s}.$$

In all simulation studies, subject-specific brain maps $X_{v}^{s}$ and $Y_{v}^{s}$ were generated from the mixed-effects models (1) and (2). For each combination of model parameters, we generated 500 independent replicates of *S* = 100 subjects’ brain maps. The bootstrap-based method and the corresponding permutation-based procedures were applied to each replicate, and mean rejection rates were computed across 500 replicates. Detailed data-generation procedures are provided in Supplementary Section S2.

### 3.1 Testing correspondence between stationary population-mean brain maps

We first compared the bootstrap-based method, the spin test, and BrainSMASH under the null hypothesis:

$$H_{0}:{\text{Corr}}_{V}\left(\alpha_{X,v},\;\alpha_{Y,v}\right)=0$$

in a setting where the population-mean brain maps, $\mu_{X}+\alpha_{X,v}$ and $\mu_{Y}+\alpha_{Y,v}$ , followed spatially stationary processes. Specifically, the population-mean maps were generated from the Gaussian random field (GRF) model designed by Burt et al. (2020) and Markello and Misic (2021). The spatial autocorrelation parameter of each map was fixed at 0.5, while the correlation between population-mean brain maps

$$\rho ={\text{Corr}}_{V}\left(\alpha_{X,v},\;\alpha_{Y,v}\right)$$

was varied over the range [−0.2,0.2]. The remaining model parameters were determined using empirical estimates from HCP cortical thickness and sulcal depth brain maps, so that the simulated subject-specific brain maps were similar to the HCP data. Additional simulation details are provided in Supplementary Section S2.1.

**Fig. 3.**
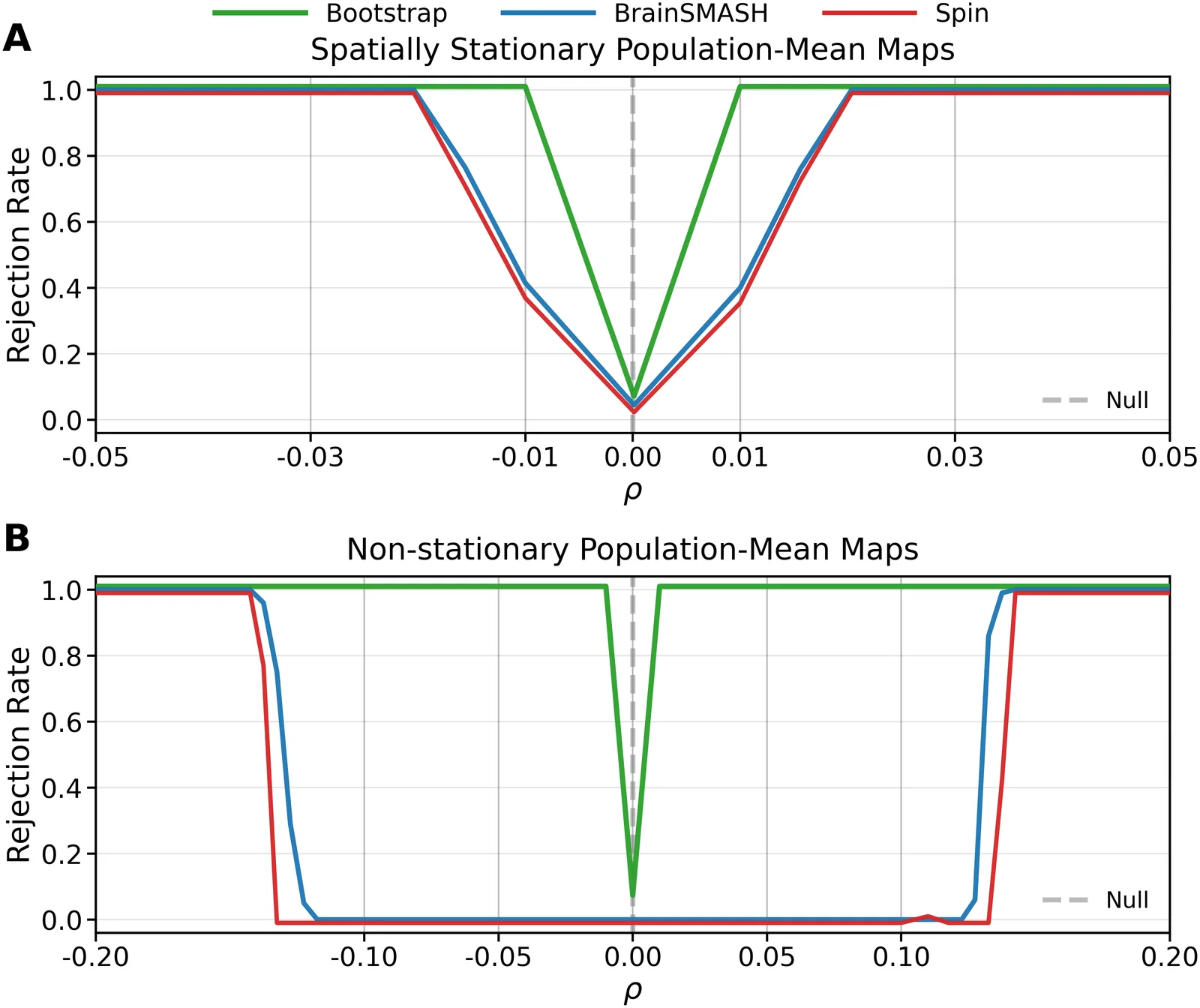
Mean rejection rates in simulation studies assessing correspondence between population-mean brain maps with *S* = 100 subjects. (A) Results for spatially stationary population-mean brain maps, shown over the narrowed range ρ∈[−0.05,0.05]. (B) Results for non-stationary population-mean brain maps. The gray dashed line indicates the null hypothesis of zero spatial correlation. Red, blue, and green curves represent mean rejection rates of the spin test, BrainSMASH, and the bootstrap-based method, respectively. Rejection rates at ρ = 0 correspond to type I error rates, while those for nonzero ρ indicate statistical power.

Figure 3A shows the mean rejection rates of the three tests over the range ρ∈[−0.05,0.05]. Rejection rates at ρ = 0 represent type I error rates, whereas rates for ρ ≠ 0 indicate statistical power. Under spatial stationarity and large *V*, the bootstrap-based method, the spin test, and BrainSMASH showed highly similar type I error rates and power.

### 3.2 Testing correspondence between non-stationary population-mean maps

We next evaluated the three methods in a setting with nonstationary population-mean brain maps designed to more closely resemble the spatial heterogeneity observed in the HCP data. All methods were tested under the same null hypothesis: $H_{0}:\rho ={\text{Corr}}_{V}\left(\alpha_{X,v},\;\alpha_{Y,v}\right)=0$ .

To generate realistic nonstationary population-mean maps, we used the empirical HCP cortical thickness and sulcal depth group-average brain maps. Specifically, the population-mean *Y* map was fixed as the group-average sulcal depth map, while the population-mean *X* map was constructed by rotating the group-average cortical thickness map. For each target correlation value ρ, we selected a rotation such that the resulting rotated cortical thickness map achieved the desired correlation with the group-average sulcal depth map. Remaining model parameters were then generated from the same empirical distributions used in Section 3.1. Additional implementation details are provided in Supplementary Section S2.2.

Figure 3B shows the rejection rates of the three methods across different values of ρ. The bootstrap-based method maintained type I error rate close to the nominal 5% level and achieved higher power than the spin test and BrainSMASH for a range of nonzero correlations. In contrast, the spin test and BrainSMASH showed low rejection rates (i.e., low power) over a range of nonzero ρ values. This pattern is consistent with the theoretical result that non-stationarity leads to overly wide null distributions for BrainSMASH and the spin test, resulting in their conservative inference and reduced statistical power.

### 3.3 Testing correspondence between subject-specific brain maps

We compared the bootstrap-based method with the SPICE test for assessing correspondence between subject-specific brain maps under models (1)–(2). We tested three null hypotheses of independence relationships:

$$H_{0}^{1}\;:\;\beta_{X}^{s}⊥\beta_{Y}^{s},\;\;\;H_{0}^{2}:\omega_{X}^{s}⊥\omega_{Y}^{s},\;\;\;\;H_{0}^{3}:\epsilon_{X,v}^{s}⊥\epsilon_{Y,v}^{s}.$$ (20)

We first set the population-mean brain maps, $\mu_{X}+\alpha_{X,v}$ and $\mu_{Y}+\alpha_{Y,v}$ , to be the group-average cortical thickness and sulcal depth maps from the HCP dataset, respectively. The interaction spatial effects, η_X, *v*_ and η_Y, *v*_, were then specified as the corresponding estimated interaction spatial patterns obtained from the cortical thickness and sulcal depth brain map data.

Next, we generated the remaining model parameters and subject-specific brain maps over a grid of parameter combinations,

$$\rho_{\beta ,\beta }\in \left[-0.5,0.5\right],\;\;\rho_{\omega ,\omega }\in \left[-0.5,0.5\right],\;\;\rho_{\epsilon ,\epsilon }\in \left[-0.2,0.2\right],$$

and $\lambda =\text{Var}\left(\eta_{X,v}\cdot \;\omega_{X}^{s}\right)/\text{Var}\left(\epsilon_{X,v}^{s}\right)\in \left\{0.5,1.0,2.0\right\}$, where λ denotes the signal-to-noise ratio (SNR). The number of subjects was fixed at *S* = 100.

For each combination $\left(\rho_{\beta ,\beta },\;\rho_{\omega ,\omega },\;\rho_{\epsilon ,\epsilon },\;\lambda \right)$, we conducted 500 independent simulation replicates, with each replicate consisting of *S* subjects’ *X* and *Y* brain maps generated from the same model. The detailed simulation setup is provided in Supplementary Section S2.3.

**Fig. 4.**
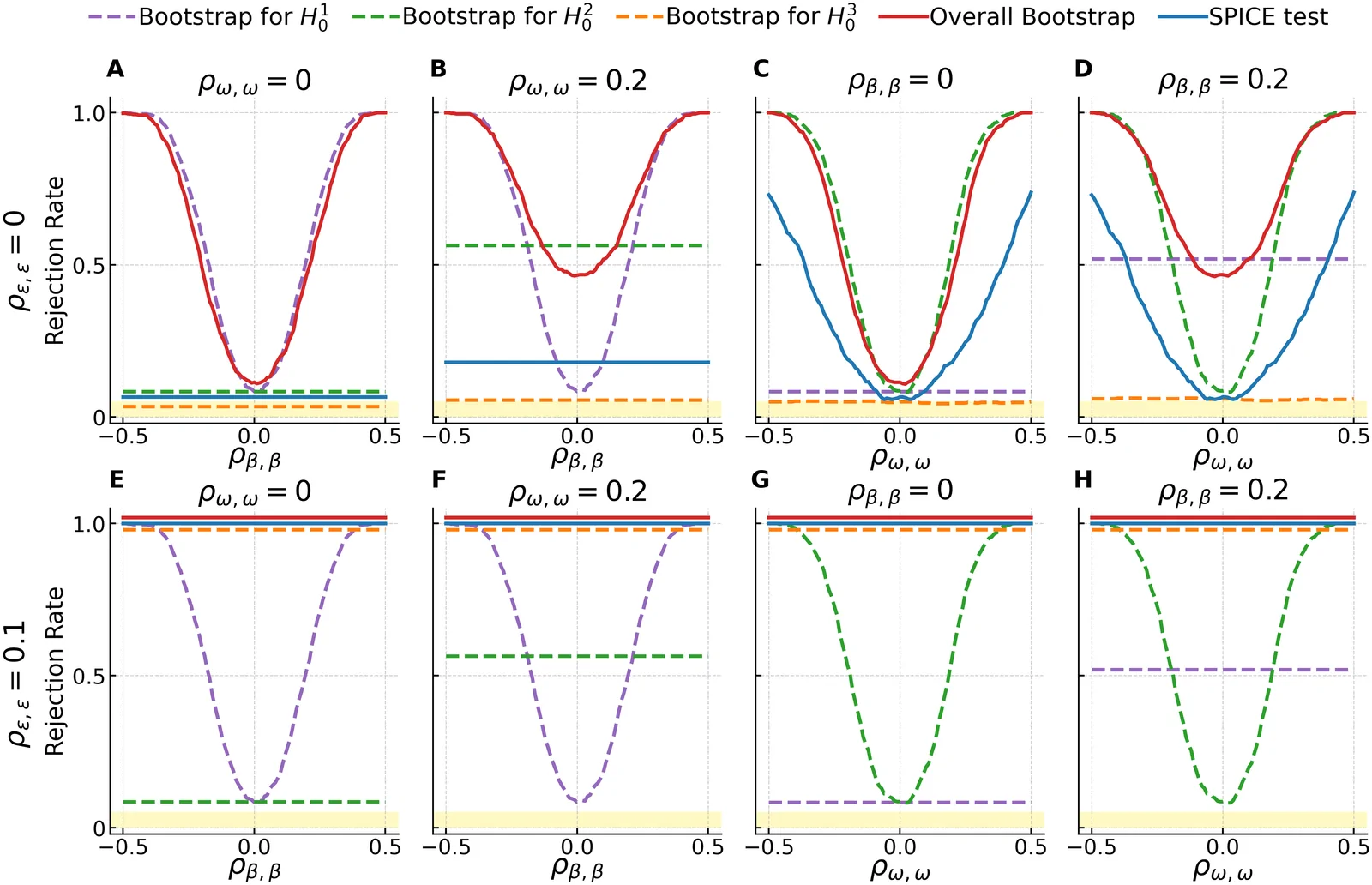
Rejection rates of the bootstrap-based method and the SPICE test in simulation studies assessing correspondence between subject-specific brain maps. The correlations ρ_β,β_, ρ_ω,ω_, and ρ_ϵ,ϵ_ were varied independently, with SNR λ = 2 and sample size *S* = 100. (A–D) Rejection rates across different combinations of ρ_β,β_ and ρ_ω,ω_ with $\rho_{\epsilon ,\epsilon }=0$ . (E–H) Rejection rates across different combinations of ρ_β,β_ and ρ_ω,ω_ with $\rho_{\epsilon ,\epsilon }=0.1$ . Purple, green, and orange curves represent rejection rates of the bootstrap-based method for separately testing independence between main subject effects ($H_{0}^{1}$), interaction subject effects ($H_{0}^{2}$), and residual terms ($H_{0}^{3}$), respectively. Red and blue curves represent rejection rates of the overall bootstrap-based method and the SPICE test. The yellow-shaded region denotes the 5% significance threshold.

Figure 4 presents rejection rates of the bootstrap-based method for testing each of the three null hypotheses in (20), the overall bootstrap-based method, and the SPICE test for different values of (ρ_β,β_, ρ_ω,ω_, ρ_ϵ,ϵ_) with λ = 2. The results revealed clear differences in the sensitivity of these approaches depending on which subject-specific sources of variability drove the correspondence.

When the correspondence was solely due to correlated main subject effects, that is, $\rho_{\omega ,\omega }\;=\rho_{\epsilon ,\epsilon }\;=0$ , the SPICE test had virtually no power in detecting the correspondence, as shown in Figure 4A. In contrast, the bootstrap-based method for testing independence between $\beta_{X}^{s}$ and $\beta_{Y}^{s}$, and the overall bootstrap-based method consistently achieved high power.

When correspondence was induced through correlated interaction subject effects (ρ_ω,ω_) while residual terms remained independent ($\rho_{\epsilon ,\epsilon }=0$ ), the bootstrap-based method again outperformed the SPICE test (Fig. 4B–D).

When the residuals in two brain maps were correlated even at a small magnitude (e.g., $\rho_{\epsilon ,\epsilon }=0.1$ ), the SPICE test, the bootstrap-based method for assessing residual independence, and the overall bootstrap-based method achieved near-perfect power (Fig. 4E–H). In these cases, the three approaches performed equivalently.

In summary, the SPICE test demonstrated high power only when correspondence arose from correlated residual terms, moderate or low power when driven by correlated interaction subject effects, and no power when driven purely by main subject effects. In contrast, the bootstrap-based method achieved strong performance across all scenarios.

Additional simulations with different SNR values λ (Supplementary Figs. S7 and S8) yielded consistent findings, further confirming the generality of these results.

## 4 Brain Map Correspondence in HCP Data

### 4.1 Correspondence between population-mean brain maps

We assessed the correspondence between the population-mean brain maps of cortical thickness and sulcal depth based on the HCP dataset. We applied the bootstrap-based method, the spin test, and BrainSMASH to brain maps of HCP subjects. All tests were conducted separately for the left and right hemispheres, since the null models of the spin test and BrainSMASH are defined on hemisphere-specific surfaces. Each test for each hemisphere was performed at the three spatial resolutions (vertex, 360 parcels, and 1,000 parcels) as defined in Section 2.3. In total, this yielded 18 analyses (three methods × two hemispheres × three resolutions) for assessing population-mean brain-map correspondence.

Results are summarized in Table 3. Across all hemispheres and resolutions, the observed correlations between group-average cortical thickness and sulcal depth brain maps were modest and positive ($\hat{\rho }$ ranging from 0.101 to 0.185). Within this range, correlations were larger at the vertex level than after parcellation ($\hat{\rho }=0.163$ –0.185 versus 0.101–0.142), consistent with the possibility that parcel averaging smooths fine-scale structural variation, particularly in sulcal depth, and thereby attenuates localized cortical thickness–sulcal depth correspondence. The bootstrap-based procedure consistently detected significantly nonzero correlations between the two types of brain maps: all 95% confidence intervals excluded zero across spatial scales and hemispheres. Supplementary Figure S9A shows the bootstrap-based distributions of the correlations for the left hemisphere, demonstrating stable positive correspondence.

**Table 3.** Inference results for correspondence between population-mean cortical thickness and sulcal depth maps using the spin test, BrainSMASH, and the bootstrap-based method.

			Permutation *p*-values		Bootstrap-Based Inference	
Atlas/Resolution	Hem.	Test Statistic ($\hat{\rho }$ )	Spin	BrainSMASH	95% CI	Decision
HCP-MMP	L	0.127	0.213	0.230	(0.123, 0.131)	Reject
(180 parcels)	R	0.142	0.155	0.192	(0.138, 0.146)	Reject
Schaefer-1000	L	0.122	0.137	0.135	(0.119, 0.125)	Reject
(500 parcels)	R	0.101	0.062	0.158	(0.098, 0.105)	Reject
Vertex-level	L	0.163	0.026*	0.048*	(0.160, 0.167)	Reject
(32k vertices)	R	0.185	0.007**	0.010*	(0.181, 0.188)	Reject

The table reports two-sided p-values from the spin test and BrainSMASH. For the bootstrap-based method, 95% confidence intervals for the correlation between the population-mean maps are provided. The null hypothesis of no correspondence is rejected by the bootstrap-based method when the confidence interval excludes zero.

* *p*-value <0.05, **p-value <0.01.

In contrast, the spin test and BrainSMASH detected significant correspondence only at the vertex-level resolution; at the 180- and 500-parcel scales, both methods yielded non-significant results (all *p*-values >0.05 ).

### 4.2 Correspondence between subject-specific brain maps

We next examined correspondence between subject-specific cortical thickness and sulcal depth brain maps using the SPICE test and the bootstrap-based method. As shown in Table 4(A), the SPICE test detected significant correspondence in both hemispheres across all spatial resolutions (the test statistic of the SPICE test $A_{0}\in \left[0.165,\;0.267\right]$, all *p*-values =0.001 ).

**Table 4.** Inference results for correspondence between subject-specific brain maps using the SPICE test and the bootstrap-based method.

Atlas	Hem.	SPICE Test	Bootstrap-Based Procedures						
				ρ_β,β_	$H_{0}^{1}$	ρ_ω,ω_	$H_{0}^{2}$	ρ_ϵ,ϵ_	$H_{0}^{3}$
		*A*_0_	p-value	95% CI	Decision	95% CI	Decision	95% CI	Decision
**(A) Cortical Thickness–Sulcal Depth**									
HCP-MMP	L	0.17	0.001*	(0.06, 0.17)	Reject	(-0.05, 0.14)	FTR	(0.30, 0.32)	Reject
(180 parcels)	R	0.18	0.001*	(0.07, 0.20)	Reject	(-0.11, 0.10)	FTR	(0.31, 0.32)	Reject
Schaefer-1000	L	0.19	0.001*	(0.10, 0.21)	Reject	(-0.05, 0.15)	FTR	(0.35, 0.36)	Reject
(500 parcels)	R	0.17	0.001*	(0.10, 0.21)	Reject	(-0.07, 0.14)	FTR	(0.37, 0.38)	Reject
Vertex-level	L	0.26	0.001*	(0.11, 0.23)	Reject	(-0.18, 0.21)	FTR	(0.38, 0.38)	Reject
(32k vertices)	R	0.27	0.001*	(0.13, 0.24)	Reject	(-0.14, 0.17)	FTR	(0.38, 0.39)	Reject
**(B) Motor Contrast–Language Contrast**									
HCP-MMP	L	-0.13	0.001*	(-0.10, 0.02)	FTR	(0.18, 0.31)	Reject	(-0.09, -0.07)	Reject
(180 parcels)	R	-0.14	0.001*	(-0.17, -0.04)	Reject	(0.21, 0.34)	Reject	(-0.10, -0.08)	Reject
Schaefer-1000	L	-0.11	0.001*	(-0.10, 0.04)	FTR	(0.17, 0.31)	Reject	(-0.09, -0.07)	Reject
(500 parcels)	R	-0.11	0.001*	(-0.16, -0.04)	Reject	(0.20, 0.33)	Reject	(-0.11, -0.09)	Reject
Vertex-level	L	-0.10	0.001*	(-0.09, 0.04)	FTR	(0.18, 0.33)	Reject	(-0.08, -0.07)	Reject
(32k vertices)	R	-0.11	0.001*	(-0.16, -0.04)	Reject	(0.22, 0.35)	Reject	(-0.09, -0.08)	Reject

$H_{0}^{1}$: $\beta_{X}^{s}⊥\beta_{Y}^{s}$, $H_{0}^{2}$: $\omega_{X}^{s}⊥\omega_{Y}^{s}$, and $H_{0}^{3}$: $\epsilon_{X,v}^{s}⊥\epsilon_{Y,v}^{s}$.

* *p*-value <0.05.

FTR = Fail to Reject. Decision indicates whether the null hypothesis is rejected (95% CI excludes zero).

The bootstrap-based procedure also identified significant correspondence, while additionally revealing which components of brain map variability contributed to the correspondence. As summarized in Table 4(A), the bootstrap results showed significant correlations between the main subject effects in the two brain maps (ρ_β,_*_β_*) and between their residuals (ρ_ϵ,ϵ_), with corresponding 95% confidence intervals excluding zero. By contrast, the interaction subject effects were not significantly correlated (ρ_ω,ω_), and the cross-term correlations between interaction subject effects and residual terms (ρ_ϵ,ω_ and ρ_ω,ϵ_) were very small in magnitude, indicating practically negligible cross-component associations (Supplementary Table S1). Supplementary Figure S9B and D displays the bootstrap sampling distributions for these correlations.

To assess whether these correspondence patterns generalize to other modalities, we repeated the analysis using motor and language contrast brain maps from the same subjects (obtained using the subjects’ task-based fMRI data). Results are given in Table 4(B). The SPICE test again detected significant correspondence across hemispheres and resolutions. However, the associations were negative ($A_{0}\in \left[-0.143,\;-0.099\right]$, p=0.001 ), opposite in direction to those observed for the structural brain maps.

The bootstrap-based analysis revealed several differences in the correspondence patterns between the two functional brain maps compared with those observed for the structural brain maps. First, the main subject effects (ρ_β,β_) showed only modest and hemisphere-specific significance, being significant in the right hemisphere but not in the left. Second, the correlation between interaction subject effects (ρ_ω,ω_) was larger and significantly positive. Third, although the correlation between residuals (ρ_ϵ,ϵ_) remained statistically significant, its magnitude was close to zero, and the cross-terms between residual terms and interaction effects remained negligible, with narrow confidence intervals (all 95% CI widths <0.01 ). Panels B and D of Supplementary Figure S10 display the corresponding bootstrap sampling distributions.

In summary, these results indicate that the sources of subject-level correspondence differ substantially across modalities. These results highlight the value of the bootstrap-based method for revealing modality-specific mechanisms of correspondence that are not identifiable with the SPICE test alone.

## 5 Discussion

### 5.1 Comparison of different tests under mixed-effects models

Using the mixed-effects models, we clarify similarities and fundamental differences among permutation-based correspondence tests. Within this framework, the spin test and BrainSMASH are shown to target correspondence between population-mean brain maps, which are captured by main spatial effects. In contrast, the SPICE test focuses on correspondence between subject-specific spatial variability across brain locations, which is characterized by brain-wide interaction subject effects and residuals.

Beyond these distinctions, the mixed-effects formulation naturally extends to the proposed bootstrap-based method, which leverages explicit model parameters describing different sources of variability in brain maps. This enables simultaneous assessment of multiple forms of correspondence between different components of brain map variability, including main spatial effects, main subject effects, interaction effects, and residuals, offering a more comprehensive and flexible inference framework than any single permutation-based test.

#### 5.1.1 Distinct conceptual assumptions regarding population-mean brain maps

Our framework clarifies a fundamental but often implicit distinction in how previous studies conceptualize population-mean brain maps and, equivalently, group-average brain maps when the number of subjects *S* is large. In the proposed mixed-effects framework, population-mean maps are treated as *fixed* characteristics of the underlying population. Even under spatial stationarity, this formulation assumes a single realized spatial pattern representing the true population mean, that is, one realization of an underlying spatial process rather than repeatedly generated random realizations. Consequently, variability in the observed correspondence statistic arises solely from finite-sample subject variability, not from randomness in the population-mean maps themselves. As *S* increases, the group-average brain maps converge to the underlying population-mean maps, and the correlation between group-average maps correspondingly converges to the true population-level correspondence. Under this framework, the bootstrap-based confidence interval for the population-mean correlation becomes increasingly narrow as *S* grows.

In contrast, several previous studies (Markello & Misic, 2021) implicitly adopted a *random population-mean* perspective by allowing population-mean maps to vary across samples and generating them independently from stochastic spatial processes in simulation studies. Under this formulation, even the population-mean maps themselves are random quantities, so the observed correspondence between maps varies across realizations of the underlying spatial process.

#### 5.1.2 Different inferential outcomes under fixed vs. random population-mean assumptions

These two perspectives lead to fundamentally different interpretations of statistical inference and, consequently, different conclusions regarding the performance of correspondence-testing methods. For example, previous studies (Markello & Misic, 2021) generated group-average brain maps directly from stationary Gaussian random fields (GRFs) with zero expected cross-map correspondence. Under strong spatial autocorrelation, independent realizations of such GRFs can nevertheless exhibit moderate or even large correlations purely by chance. Within the random population-mean framework, these correlations are interpreted as false positives because the underlying stochastic process has zero expected correspondence.

Under the fixed-effects interpretation adopted in our framework, however, each realized population-mean map is regarded as the true spatial structure of a population. Therefore, once a realization is observed, a nonzero correlation between the resulting population-mean maps represents a genuine fixed relationship between the maps rather than a spurious one. In this setting, rejecting the null hypothesis corresponds to detecting a true positive association between the realized population-level spatial patterns.

To illustrate this distinction, we performed additional simulations (Supplementary Section S3.2; Supplementary Fig S6) using stationary GRFs with a high spatial autocorrelation (=3.0 ) and zero expected correspondence. Each GRF realization was treated as fixed population-mean brain maps, from which 500 independent replicates of *S* subjects’ brain maps were generated. For realizations exhibiting strong correlations between population-mean brain maps, all three methods (the spin test, BrainSMASH, and the bootstrap-based method) rejected the null hypothesis. Under our framework, these are true positives, whereas under the random population-mean perspective, they are classified as false positives.

#### 5.1.3 Theoretical and empirical support for fixed population means

Both theoretical and empirical evidence support the interpretation of population-mean brain maps as fixed quantities. Theoretically, by the law of large numbers, the sample mean of subject-level brain maps at each spatial location converges to a constant as the number of subjects increases (Eq. (3)). Empirically, analyses of the HCP dataset demonstrate that group-average brain maps rapidly stabilize with increasing sample size (Supplementary Fig. S4). Together, these results suggest that population-mean brain maps behave as stable characteristics of the underlying population rather than random realizations repeatedly generated from a spatial stochastic process.

#### 5.1.4 The effects of spatial resolution and autocorrelation

Under the assumption of fixed population-mean brain maps and with sufficiently large *S*, the spin test and BrainSMASH tend to produce conservative inference, as their permutation-based null distributions remain wider than the bootstrap-based sampling distribution, encompassing a range of nonzero correlations.

Even when population-mean brain maps arise from stationary processes, the behavior of permutation-based methods depends critically on spatial resolution (the number of brain locations *V*). When *V* is large, the permutation-based null distributions of the spin test and BrainSMASH contract toward zero, and their inferential outcomes are similar to those of the bootstrap-based method. However, when *V* is small, the permutation-derived correlations, ${\text{Corr}}_{V}\left({\bar{X}}_{\pi \left(v\right)},\;{\bar{Y}}_{v}\right)$ for the spin test and ${\text{Corr}}_{V}\left({\overset{⌣}{X}}_{v},{\bar{Y}}_{v}\right)$ for BrainSMASH, exhibit substantial variability, even with large *S*. This inflated variability widens the null distributions around zero and substantially reduces power.

Simulation analyses (Supplementary Fig. S5A) confirm this behavior: when the same population-mean brain maps generated from stationary GRFs were analyzed at a lower spatial resolution (e.g., reducing from 32k vertices to 500 parcels), the power of both the spin test and BrainSMASH dropped to near zero across a range of nonzero correlations. Analyses of real cortical thickness and sulcal depth data showed the same pattern: both the spin test and BrainSMASH detected significant correspondence only at the high spatial resolution (32k vertices; Table 3).

Spatial autocorrelation further magnifies these issues. Supplementary Figure S5B demonstrates that even at a high resolution of 32k vertices, increasing spatial autocorrelation from 0.5 to 3 dramatically reduces power for the spin test and BrainSMASH. Stronger spatial dependency requires a much larger *V* for permutation-based null distributions to concentrate near zero.

In practice, real brain maps rarely exhibit spatial stationarity, as evidenced by their highly heterogeneous regional variograms (Supplementary Fig. S3). Such nonstationarity prevents permutation-derived surrogate maps from preserving the true spatial structure, causing the null distributions of the spin test and BrainSMASH to broaden and resulting in conservative inference.

#### 5.1.5 The SPICE test

The SPICE test targets a different type of correspondence: between subject-specific rather than population-mean brain maps. It employs a distinct test statistic: the average correlation between individual subjects’ *X* and *Y* maps. Within the mixed-effects framework, we show that SPICE implicitly tests independence of subject-specific spatial variability across the two maps, a condition that encompasses multiple independence requirements: (1) independence between brain-wide interaction subject effects of the two maps, (2) independence between residual terms across maps, and (3) independence between residuals and interaction effects across maps. Thus, significant correspondence detected by the SPICE test can originate from several distinct sources of subject-specific variability.

Comparisons of correspondence patterns between structural and functional brain maps further illustrate that identical SPICE test results can arise from different underlying sources of subject-specific brain map correspondence. In other words, a significant SPICE result does not reveal which components of variability, interaction subject effects, residuals, or cross-component dependencies drive the observed association. These findings underscore the importance of explicitly modeling and disentangling different sources of variability in brain maps.

### 5.2 Advantages of the bootstrap-based method

Building on the distinctions revealed by the mixed-effects models, the proposed bootstrap-based inference framework offers several conceptual and practical advantages over permutation tests.

First, the bootstrap procedures are explicitly grounded in the mixed-effects formulation, which decomposes total brain map variability into distinct components: population-mean spatial variability, variation across subjects, their interactions, and residual terms. This formulation allows the bootstrap-based method to directly target each source of variability and to assess multiple compositions of correspondence within a single, coherent resampling scheme. Specifically, from the same set of bootstrap samples, one can obtain multiple correlation estimates, such as ${\hat{\rho }}^{\left(b\right)}$, ${\hat{\rho }}_{\beta ,\beta }^{\left(b\right)}$, ${\hat{\rho }}_{\omega ,\omega }^{\left(b\right)}$, ${\hat{\rho }}_{\epsilon ,\epsilon }^{\left(b\right)}$, ${\hat{\rho }}_{\epsilon ,\omega }^{\left(b\right)}$, and ${\hat{\rho }}_{\omega ,\epsilon }^{\left(b\right)}$, each reflecting correspondence between specific components of brain map variability. By examining these different components separately, the bootstrap-based method provides a more detailed understanding of various biological and imaging methodological factors driving observed correspondence between brain maps, which no single permutation test can offer.

Second, unlike permutation tests that focus solely on whether an observed statistic deviates from a null distribution, the bootstrap-based method quantifies the sampling variability of correspondence estimates. This facilitates inference not only on the presence of correspondence but also on its magnitude and uncertainty, providing richer and more nuanced insight than the binary reject-or-not-reject outcomes typical of permutation tests.

Third, the proposed mixed-effects framework offers substantial flexibility for characterizing spatial patterns in brain maps, as the parameters $\mu_{X}\;+\alpha_{X,v}$ , $\mu_{Y}\;+\alpha_{Y,v}$ , η_X,_*_v_*, and η_Y,_*_v_* are allowed to vary freely across spatial locations *v*. In addition, the bootstrap-based inference procedure is fully nonparametric and does not rely on explicit assumptions about the spatial distribution or dependence structure of residuals. As a result, the bootstrap-based method is robust and broadly applicable across imaging modalities, spatial resolutions, and patterns of spatial autocorrelation.

To empirically demonstrate this robustness, we conducted an additional simulation study on the fs_LR 32k cortical surface (see Supplementary Section S3.8). Data were generated under the null hypothesis of zero cross-map correlations, while residuals were simulated from GRFs with varying levels of spatial autocorrelation, controlled by $\alpha_{\text{GRF}}\in \left\{0,\;1.5,\;3.0\right\}$. Across all settings, the empirical false positive rates remained close to the nominal 5% level (Supplementary Table S3), confirming that the proposed method maintains valid inference even under strong spatial dependence.

### 5.3 Structural and functional brain map correspondence

While both structural (cortical thickness and sulcal depth) and functional (language and motor contrast) brain maps show significant subject-level correspondence, the underlying components driving these associations differ markedly in their biological and methodological origins.

First, the correspondence between main subject effects (ρ_β,β_) showed distinct patterns across modalities. For the structural brain maps, ρ_β,_*_β_* was significant in both hemispheres, indicating a strong association between brain-wide cortical thickness and sulcal depth. This finding aligns with the well-established notion that both measures reflect common aspects of cortical morphology and a consistent topographical organization shared across individuals (Tallinen et al., 2014). However, for the language and motor contrast maps, ρ_β,β_ captures across-subject coupling in average task-evoked activation rather than shared cortical morphology. Direct bootstrap comparisons showed that this coupling differed between hemispheres across spatial resolutions (Supplementary Table S2), consistent with known hemispheric differences in task-fMRI activation patterns (Barch et al., 2013).

Second, the significant positive correlation between interaction subject effects (ρ_ω,ω_) in the functional brain maps indicates that individuals consistently express the dominant spatial deviation patterns across tasks. This interpretation is consistent with the “connectome fingerprint” framework (Finn et al., 2015), which shows that functional networks, particularly in higher-order association cortices, exhibit reproducible within-subject organization and variability across individuals.

Finally, the strong correlation between residuals in individual subjects’ cortical thickness and sulcal depth brain maps is likely due to the remaining spatial variability unexplained by the interaction effects. The interaction effects account for subject-specific variability only along the most dominant spatial variability patterns. Therefore, residuals contain subject-specific spatial variability in the different spatial patterns that are potentially strongly correlated between different maps. Future research can be focused on modeling various spatial patterns that individual brain maps can demonstrate.

Overall, by disentangling different sources of brain map variability, the bootstrap-based method reveals the specific components that contribute to structural and functional correspondence. This decomposition enables researchers to distinguish biologically meaningful associations from those driven by methodological or acquisition-related factors, providing a clearer interpretation of brain map correspondence.

### 5.4 Future research

The current manuscript focuses on mixed-effects models for assessing correspondence between two maps defined on the same spatial domain. Nevertheless, the proposed framework can be naturally extended to settings in which the two maps are defined on different spaces or have different spatial resolutions, such as white matter tract measures defined on white matter skeletons versus cortical thickness or task-fMRI maps defined on the cortical surface. In these settings, the number of spatial locations or regions may differ across modalities, making direct voxel-wise or region-wise correlation inappropriate.

To address this challenge, alternative similarity measures between population-mean brain maps can be incorporated into the proposed framework. For example, optimal transport-based distances, including Wasserstein and Gromov–Wasserstein (GW) distances (Mémoli, 2011; Thual et al., 2022), provide principled approaches for quantifying correspondence between maps defined on different domains by accounting for their underlying geometric structures. Importantly, the subject-specific components of the proposed mixed-effects framework, such as ρ_β,β_ and ρ_ω,ω_, remain well defined under these extensions because they characterize between-subject variability rather than relying on one-to-one spatial matching. Consequently, the proposed bootstrap inference procedures can still be applied to obtain confidence intervals and hypothesis tests for these generalized measures of brain map correspondence.

Despite its flexibility, the current model captures subject-specific spatial variability through a single dominant interaction term, $\eta_{X,v}\cdot \;\omega_{X}^{s}$ and $\eta_{Y,v}\cdot \;\omega_{Y}^{s}$. This formulation reflects variability along the most prominent spatial pattern across subjects but may not fully capture more complex spatial heterogeneity. Consequently, additional subject-specific spatial variation may be absorbed into the residuals, which could partly explain the observed residual correlations between cortical thickness and sulcal depth maps.

The current manuscript presents a general modeling framework for whole-brain maps. Several extensions could be developed to more comprehensively characterize subject-specific spatial variability and spatially localized correspondence patterns. First, it is possible that only specific subsets of voxels or brain regions in one modality are associated with localized regions in another modality. To accommodate such spatial specificity, the proposed framework could be extended by introducing spatial group labels or region-specific components, allowing model parameters and correspondence strengths to vary across regions. Second, multi-factor low-rank models could incorporate multiple spatial components, enabling richer representations of inter-subject variability. Such extensions would naturally allow component-specific correspondence analyses, where different spatial components capture associations localized to distinct subsets of brain regions. Third, brain maps demonstrate spatially varying covariance structures, as shown in Supplementary Figure S11. Gaussian process-based models could be used to flexibly model these complex and localized spatial dependencies while maintaining computational tractability (Mejia et al., 2020; Whiteman et al., 2024). Fourth, the framework can also be extended to incorporate subject-level covariates. For example, subject-specific parameters, such as $\beta_{X}^{s}$, $\beta_{Y}^{s}$, $\omega_{X}^{s}$, and $\omega_{Y}^{s}$, may be modeled as functions of demographic or clinical covariates, including age, sex, or disease status. Finally, the covariance structure itself may depend on subject-specific effects. For example, Supplementary Figure S12 suggests potential associations between residual variability and the main subject effects, motivating future models in which covariance structures vary as functions of subject-level characteristics.

## Supplementary Material

Supplementary Material

## Data Availability

The open access HCP-S1200 data can be downloaded from the data management platform ConnectomeDB: https://db.humanconnectome.org upon signing up for an account. The code for the analyses presented in this paper is included as a compressed file in the supplementary materials and is also available on GitHub (https://github.com/qcwang77/brainmap-bootstrap).
