# Supplementary material for: Clarifying and extending permutation tests on brain map correspondence through mixed-effects modeling
Source: Imaging Neurosci (Camb). 2026 Sep 8;4:IMAG.a.1350. doi: 10.1162/IMAG.a.1350 (PMC13556793; doi:10.1162/IMAG.a.1350)
Supplement: Supplementary file 1 [file IMAG.a.1350_supp.pdf]

Supplementary Material for Clarifying and Extending Permutation Tests on Brain Map Correspondence Through Mixed-Effects Modeling

Qiaochu Wang,[1†] Lingyi Peng,[2†] Thomas E. Nichols,[3]
Xu Zou,[1] Yaotian Wang,[4] Jie He,[1] Yuexin Zhang,[1]
Dana L. Tudorascu,[2] Diego Szczupak,[5] Lauren Schaeffer,[5]
Emily S. Rothwell,[5] Laurel Dieckhaus,[5] Stacey J. Sukoff Rizzo,[5]
Gregory W. Carter,[6] Afonso C. Silva,[5] and Tingting Zhang[1*]

[1]Department of Statistics, University of Pittsburgh,
230 S Bouquet Street, Pittsburgh, PA 15260, USA

[2]Department of Biostatistics and Health Data Science, University of Pittsburgh,
130 De Soto Street, Pittsburgh, PA 15261, USA

[3]Oxford Big Data Institute, Nuffield Department of Population Health,
University of Oxford, Oxford, OX3 7LF, UK

[4]Department of Biostatistics and Bioinformatics, Emory University,
1518 Clifton Rd. NE, Atlanta, GA 30322, USA

[5]Department of Neurobiology, University of Pittsburgh,
200 Lothrop Street, Pittsburgh, PA 15213, USA

[6]The Jackson Laboratory, 600 Main Street, Bar Harbor, ME 04609, USA

[†]Lingyi Peng and Qiaochu Wang are equally contributing authors

[*]Corresponding author: Tingting Zhang, 1826 Wesley W. Posvar Hall, Room 108,
University of Pittsburgh, Pittsburgh, PA 15260, USA. Email: tiz67@pitt.edu

July 23, 2026

1 Supplementary Materials and Methods

1.1 Multi-Resolution Brain Maps

Figure 1 of the main text displays group-average brain maps at vertex-level resolution to illustrate the four modalities analyzed in this study. Supplementary Figure S1 complements this by presenting group-average maps ($S = 900$ subjects) across all three spatial resolutions examined: HCP-MMP (180 parcels per hemisphere), Schaefer-1000 (500 parcels per hemisphere), and vertex-level (32k vertices per hemisphere). For the four modalities, parcel-level maps were computed by averaging vertex values within each atlas-defined parcel.

As detailed in Section 4 of the main text, we analyzed the HCP brain maps separately at each of the three spatial resolutions. This allowed us to evaluate the sensitivity of the correspondence analyses to spatial resolution and to assess the robustness and consistency of the inference results across different levels of spatial resolution.

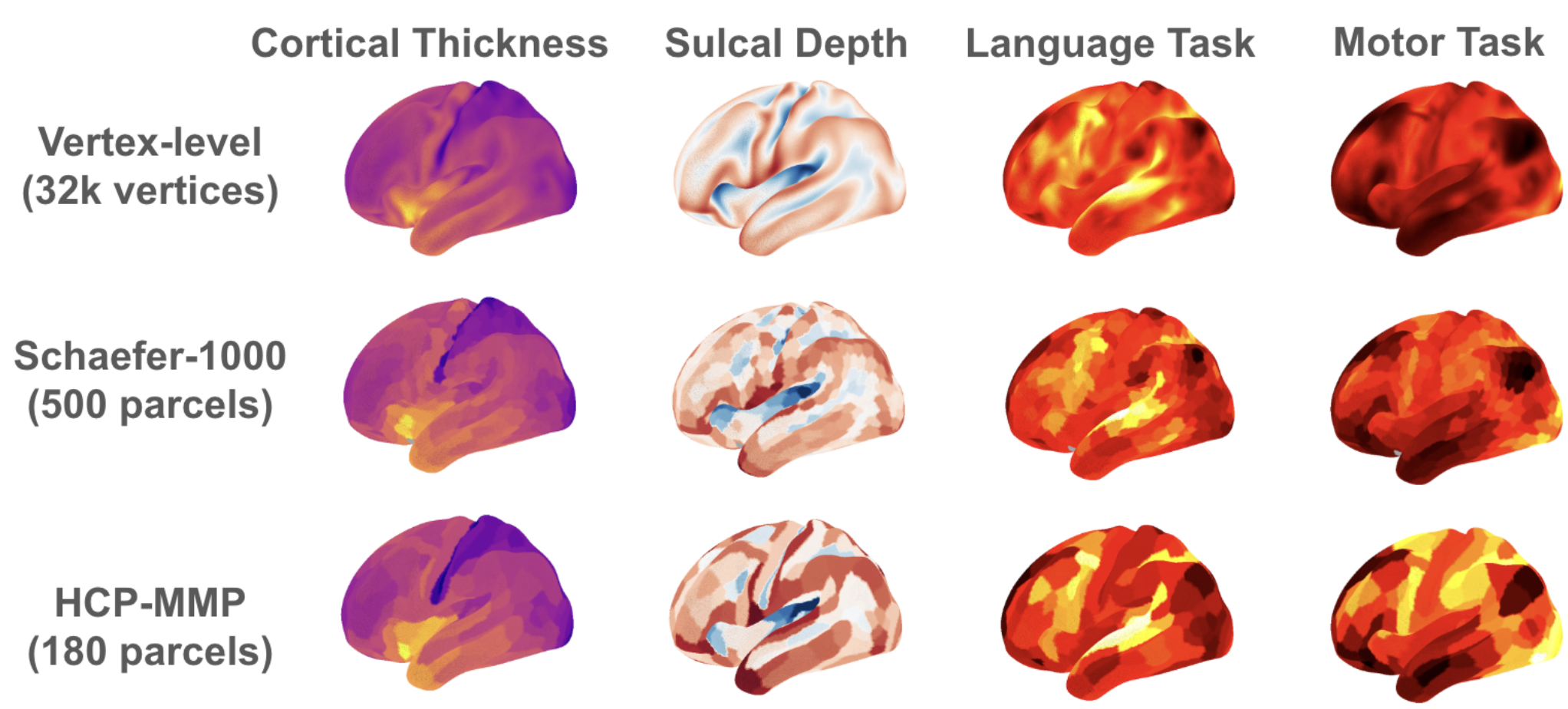

Supplementary Figure S1: Group-average brain maps in different spatial resolutions. Each row shows four brain maps (cortical thickness, sulcal depth, language task, and motor task). Each column displays one brain map in the three spatial resolutions analyzed: HCP-MMP (180 parcels), Schaefer-1000 (500 parcels), and vertex-level (32k vertices). All brain maps were obtained by averaging $S = 900$ subjects' brain maps and are displayed for the left hemisphere in the lateral view.

1.2 BrainSMASH Surrogate Map Validation

To evaluate whether BrainSMASH surrogate maps adequately preserve the spatial autocorrelation of target brain maps from the HCP cohort, we computed empirical variograms as functions of cortical

geodesic distance and compared them with the envelopes derived from $K = 1,000$ surrogate maps' variograms, following the BrainSMASH workflow (Burt et al., 2020). Supplementary Figure S2 displays these comparisons for each hemisphere and spatial resolution.

Across all hemispheres and spatial resolutions, the empirical variograms fall within or closely align with the surrogate envelopes, indicating that BrainSMASH surrogate maps successfully replicate the spatial dependence structure of target brain maps.

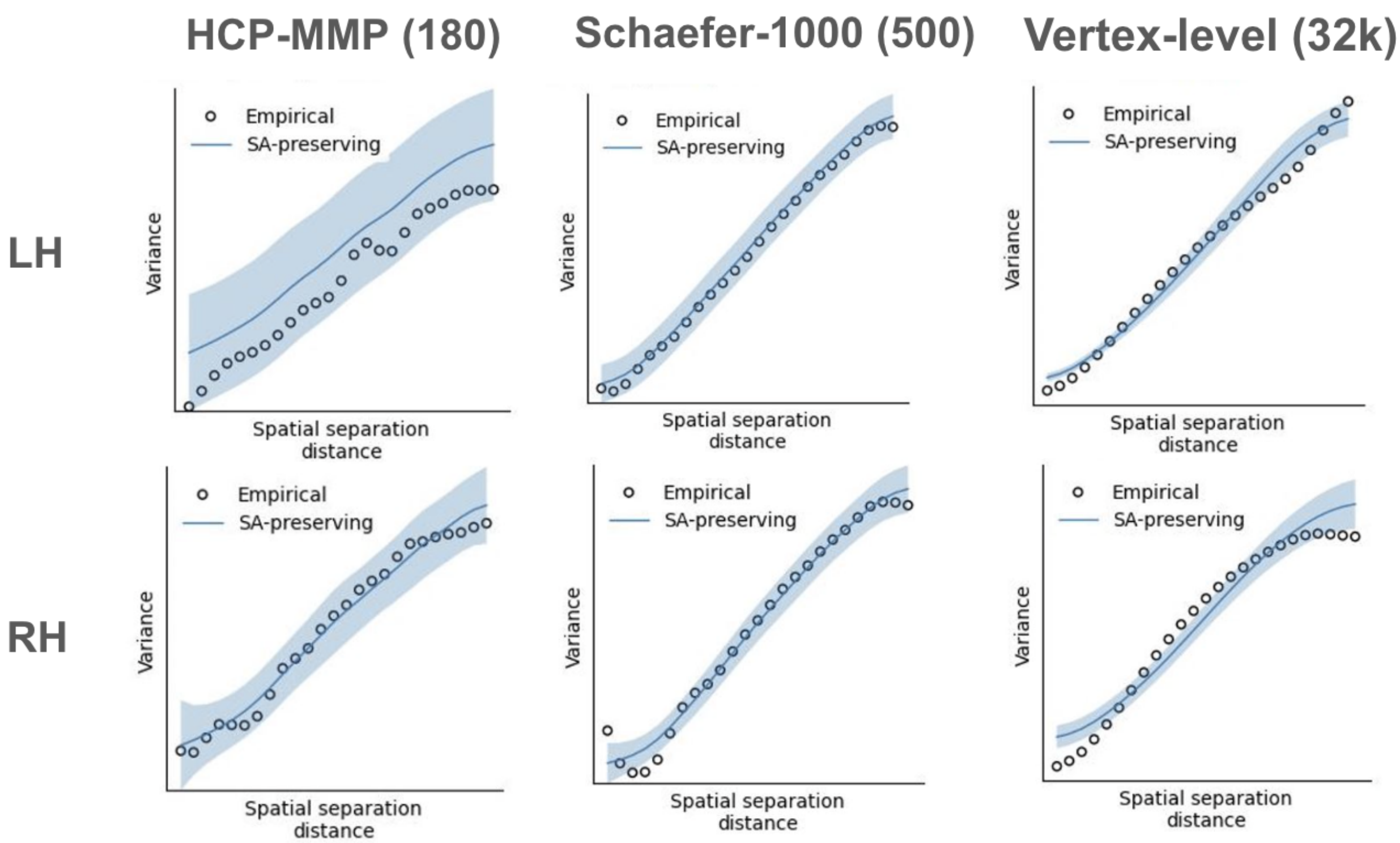

Supplementary Figure S2: Variogram envelope validation for BrainSMASH surrogate maps. Each panel shows the empirical variogram (black circles) and the envelope of variograms obtained from 1,000 BrainSMASH surrogate maps (blue shaded region) as functions of cortical geodesic distance. The upper row shows comparison results for the left hemisphere (LH) in three different spatial resolutions. The lower row shows comparison results for the right hemisphere (RH) in three different spatial resolutions.

1.3 Violation of Spatial Stationarity in Real Data

The null models used in the spin test and BrainSMASH rely on the assumption of spatial stationarity (Burt et al., 2020; Viladomat et al., 2014), which posits that the spatial correlation between any two brain regions depends solely on the geodesic distance between them, independent of their specific locations.

To test the validity of this assumption in real data, we employed a local stationarity diagnostic using

point variograms. For a given cortical map, a point variogram $\gamma_v(h)$ is computed relative to a single seed vertex v, measuring the average squared difference between the value at v and all other vertices as a function of geodesic distance h. If the map is stationary, $\gamma_v(h)$ should be identical across all seed vertices v.

We computed kernel-smoothed point variograms for representative seed vertices on the left-hemisphere cortical thickness brain map (Supplementary Figure S3). As shown in the figure, the variograms exhibit substantial heterogeneity across seed locations. The discrepancies between curves frequently exceed the 95% bootstrap bands (computed across subjects), indicating that the spatial dependence structure varies significantly across cortical regions. This location-dependent variability provides direct empirical evidence that the assumption of spatial stationarity can be violated in real data.

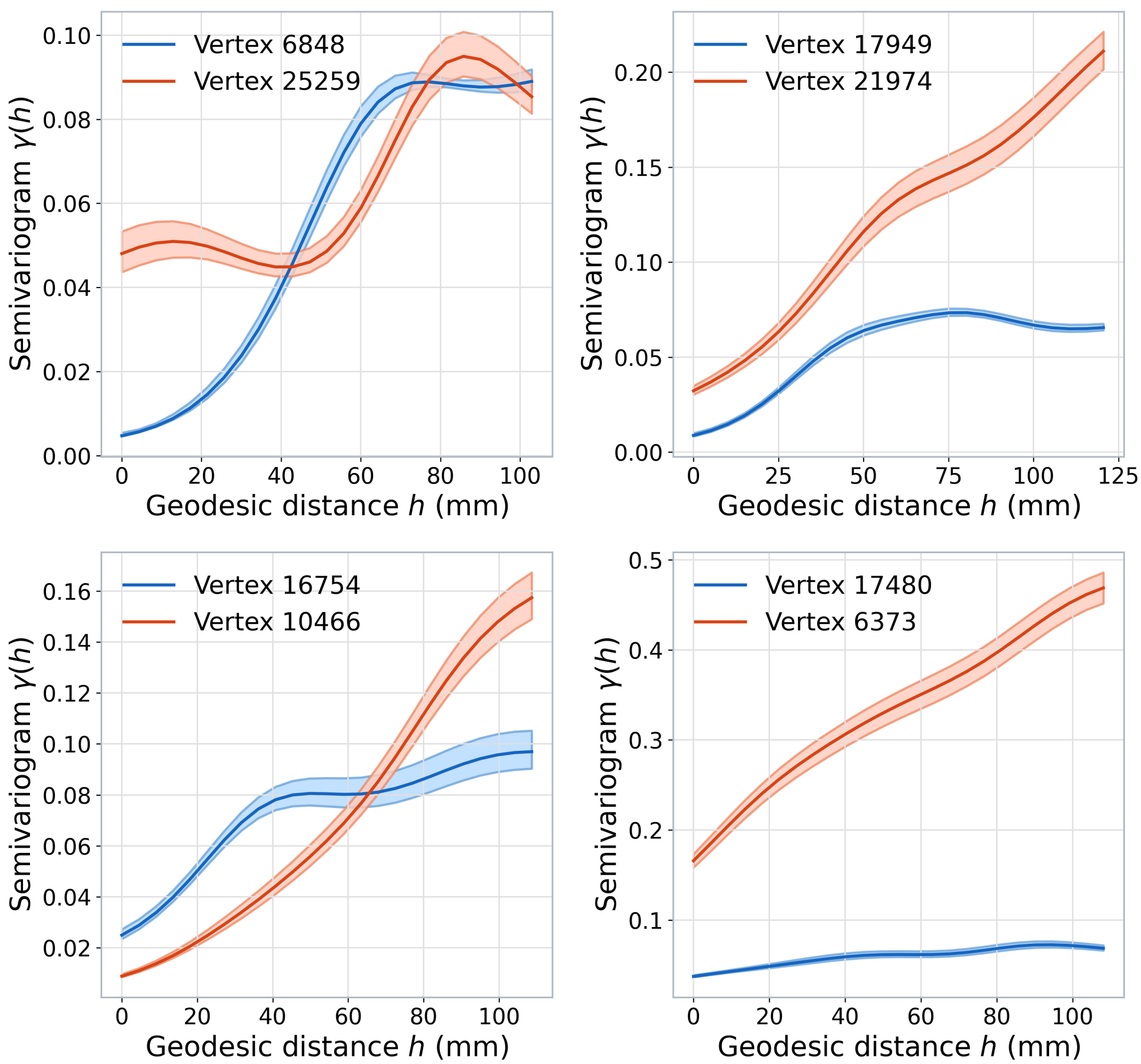

Supplementary Figure S3: Local stationarity diagnostics for cortical thickness brain maps using point variograms. Each panel displays kernel-smoothed variograms, $\gamma_v(h)$, for two representative cortical seed locations on the left hemisphere, plotted as a function of geodesic distance h (mm). Shaded ribbons indicate pointwise 95% bootstrap confidence intervals.

1.4 Empirical Validation of Fixed Population-Mean Brain Maps

We used HCP data to empirically validate the assumption that main spatial effects ($\alpha_{X,v}$ and $\alpha_{Y,v}$) and population-mean brain maps ($\mu_X + \alpha_{X,v}$ and $\mu_Y + \alpha_{Y,v}$) are fixed across study samples. Specifically, we demonstrated that group-average brain maps converge to stable spatial patterns as the sample size increases.

To assess convergence, we computed correlations between group-average brain maps obtained from two non-overlapping groups of subjects within the same modality (e.g., cortical thickness). For each subset size $n \in \{25, 50, 75, 100, 150, 200, 250, 300, 350, 400, 450\}$, we randomly drew two disjoint groups of n subjects from the full HCP cohort and calculated the Pearson correlation between their corresponding group-average brain maps. This resampling procedure was repeated 1,000 times for each n to obtain 1,000 correlations between group-average brain maps of non-overlapping subject groups.

As shown in Figure S4, correlations between group-average brain maps increased rapidly with sample size. Even with $n = 25$, correlations exceeded 0.9, and they approached 1.0 as n increases. When $n \geq 300$, the average correlation surpassed 0.99 for both cortical thickness and sulcal depth maps across all spatial resolutions (vertex-level, MMP-360, and Schaefer-1000). These results demonstrate that group-average brain maps quickly converge to highly stable spatial patterns and support treating the realized population-mean maps as stable targets of inference.

2 Supplementary Simulation Studies

This section explains detailed data-generation procedures used in the simulation studies reported in Section 3 of the main text. All simulations were conducted under the mixed-effects models in Eqs. (1)–(2). For each setting, we generated 500 independent simulation replicates with $S = 100$ subjects, unless otherwise noted. All tests were performed at the nominal 5% significance level.

2.1 Stationary Population-Mean Brain Maps

This simulation evaluated correspondence between population-mean brain maps under spatial stationarity. The bootstrap-based method, the spin test, and BrainSMASH were compared under the null hypothesis

$$H_0 : \mathrm{Corr}_{\mathcal{V}}(\alpha_{X,v}, \alpha_{Y,v}) = 0.$$

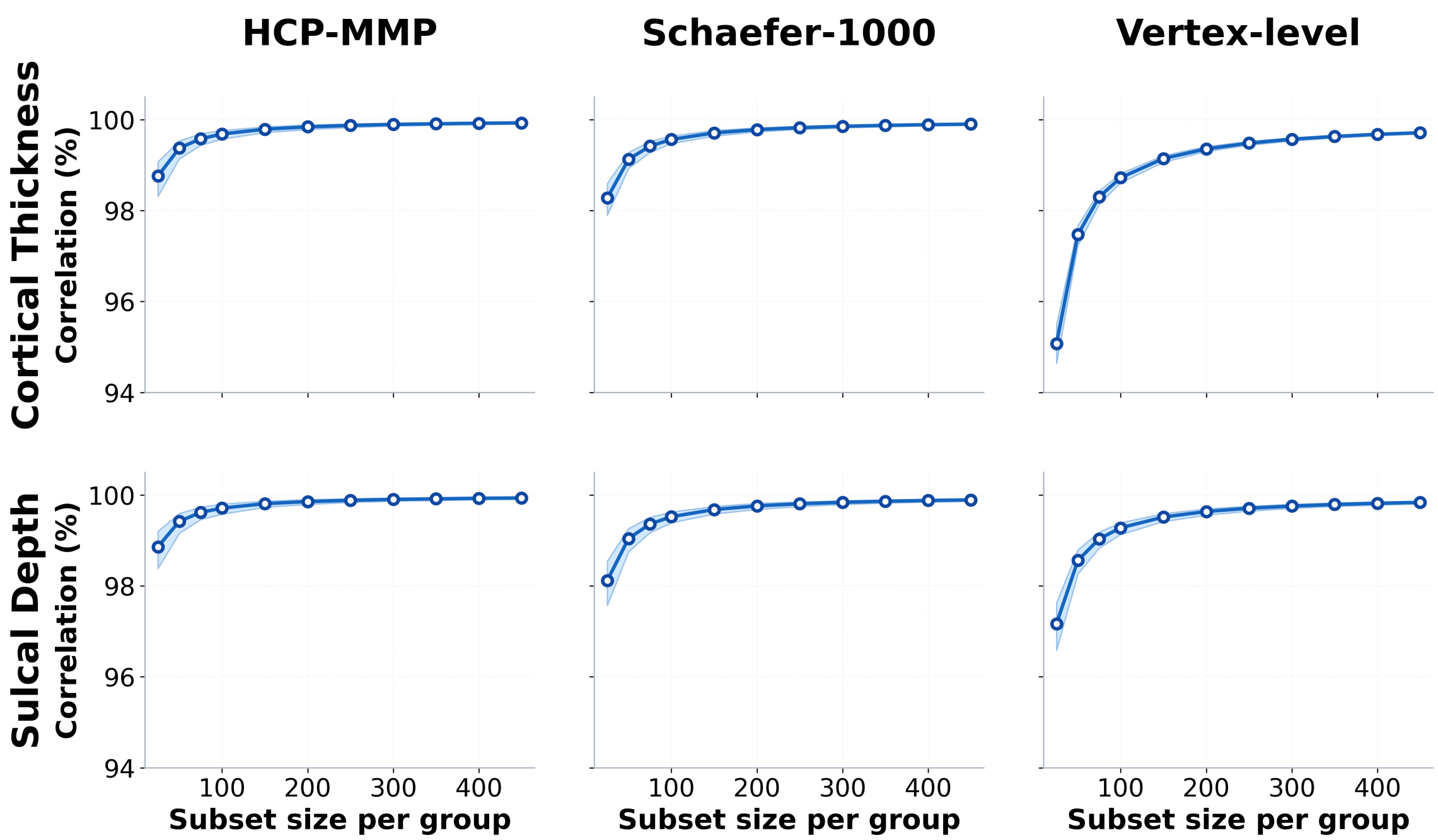

Supplementary Figure S4: Correlations between group-average brain maps from non-overlapping subject groups as a function of group size. The upper panel shows the correlations for cortical thickness brain maps in three different spatial resolutions, and the lower panel shows the correlations for sulcal depth brain maps in three spatial resolutions. For each group size, two non-overlapping subject groups were independently sampled 1,000 times, and the Pearson correlation between their corresponding group-average maps was computed. Solid lines indicate mean correlations across repetitions, and shaded areas represent 95% confidence intervals.

We generated spatially stationary population-mean maps using the GRF-based model of Burt et al. (2020) and Markello and Misic (2021). The within-map spatial autocorrelation was fixed at 0.5, and the between-map population-mean correlation ρ was varied over $[-0.2, 0.2]$, where

$$\rho = \mathrm{Corr}_{\mathcal{V}}(\alpha_{X,v}, \alpha_{Y,v}).$$

For each model-parameter setting indexed by the target correlation ρ, we generated 500 independent replicates, each containing $S = 100$ subjects with paired X and Y brain maps at vertex-level resolution.

First, population-mean X and Y brain maps, $\mu_X^\ell + \alpha_{X,v}^\ell$ and $\mu_Y^\ell + \alpha_{Y,v}^\ell$, were generated as spatially stationary GRFs with spatial autocorrelation 0.5 and cross-map correlation ρ.

Second, the interaction spatial effects were fixed at empirical estimates from the HCP cortical thickness and sulcal depth maps:

$$\eta_{X,v} = \hat{\eta}_{X,v}, \qquad \eta_{Y,v} = \hat{\eta}_{Y,v}.$$

Third, for each subject $s = 1, \ldots, S$, the subject-specific effects

$$(\beta_X^{\ell,s}, \omega_X^{\ell,s}, \beta_Y^{\ell,s}, \omega_Y^{\ell,s})$$

were independently drawn from the empirical distribution of

$$(\hat{\beta}_X^s, \hat{\omega}_X^s, \hat{\beta}_Y^s, \hat{\omega}_Y^s)$$

estimated from the HCP cohort.

Fourth, for each location v, residual terms $\epsilon_{X,v}^{\ell,s}$ and $\epsilon_{Y,v}^{\ell,s}$ were sampled independently from the empirical distributions

$$\{\hat{\epsilon}_{X,v}^s\}_{s=1}^S \quad \text{and} \quad \{\hat{\epsilon}_{Y,v}^s\}_{s=1}^S,$$

respectively.

Finally, the simulated brain maps for subject s were computed as

$$X_v^{\ell,s} = \mu_X^\ell + \alpha_{X,v}^\ell + \beta_X^{\ell,s} + \hat{\eta}_{X,v}\omega_X^{\ell,s} + \epsilon_{X,v}^{\ell,s},$$

$$Y_v^{\ell,s} = \mu_Y^\ell + \alpha_{Y,v}^\ell + \beta_Y^{\ell,s} + \hat{\eta}_{Y,v}\omega_Y^{\ell,s} + \epsilon_{Y,v}^{\ell,s}.$$

For each replicate, we applied the spin test, BrainSMASH, and the bootstrap-based method and recorded

whether each method rejected the null hypothesis. Mean rejection rates across replicates are reported in Figure 3.A of the main text.

2.2 Non-Stationary Population-Mean Brain Maps

This simulation evaluated correspondence between non-stationary population-mean brain maps. The goal was to create population-mean maps with empirical spatial structures while varying their correlation

$$\rho = \mathrm{Corr}_{\mathcal{V}}(\alpha_{X,v}, \alpha_{Y,v}).$$

All methods were tested under the same null hypothesis:

$$H_0 : \mathrm{Corr}_{\mathcal{V}}(\alpha_{X,v}, \alpha_{Y,v}) = 0.$$

For each specified ρ, we generated 500 independent simulation replicates. The population-mean Y map was fixed as the group-average sulcal depth map from the HCP dataset:

$$\mu_Y + \alpha_{Y,v} = \bar{Y}_v.$$

The interaction spatial effect for Y was fixed at its empirical estimate:

$$\eta_{Y,v} = \hat{\eta}_{Y,v}.$$

The population-mean X map was created by rotating the group-average cortical thickness map $\bar{X}_v$. Let π^ρ denote a rotation-induced permutation selected so that

$$\mathrm{Corr}_{\mathcal{V}}(\bar{X}_{\pi^\rho(v)}, \bar{Y}_v) = \rho.$$

We then set

$$\mu_X + \alpha_{X,v} = \bar{X}_{\pi^\rho(v)}, \qquad \eta_{X,v} = \hat{\eta}_{X,\pi^\rho(v)}.$$

For each replicate $\ell = 1, \ldots, 500$, subject-specific random effects and residual terms were sampled using the same empirical procedures described in Supplementary Section 2.1. Specifically,

$$(\beta_X^{\ell,s}, \omega_X^{\ell,s}, \beta_Y^{\ell,s}, \omega_Y^{\ell,s})$$

were drawn from the empirical distribution of the estimated subject effects, and $\epsilon_{X,v}^{\ell,s}$ and $\epsilon_{Y,v}^{\ell,s}$ were sampled from the empirical residual distributions at each location.

The simulated brain maps were then computed as

$$X_v^{\ell,s} = \mu_X + \alpha_{X,v} + \beta_X^{\ell,s} + \eta_{X,v}\omega_X^{\ell,s} + \epsilon_{X,v}^{\ell,s},$$

$$Y_v^{\ell,s} = \mu_Y + \alpha_{Y,v} + \beta_Y^{\ell,s} + \eta_{Y,v}\omega_Y^{\ell,s} + \epsilon_{Y,v}^{\ell,s}.$$

For each replicate, we applied the spin test, BrainSMASH, and the bootstrap-based method and recorded rejection indicators. Mean rejection rates are reported in Figure 3.B of the main text.

2.3 Subject-Specific Brain Maps Correspondence

This simulation compared the bootstrap-based method with the SPICE test for detecting correspondence between subject-specific brain maps under the mixed-effects models in Eqs. (1)–(2). We first set the population-mean brain maps $(\mu_X + \alpha_{X,v},\ \mu_Y + \alpha_{Y,v})$ to be the group-average cortical thickness and sulcal depth brain maps from the HCP dataset, respectively. We then set the interaction spatial effects ($\eta_{X,v}$, $\eta_{Y,v}$) equal to the estimated interaction spatial effects in cortical thickness and sulcal depth brain maps, respectively. To evaluate correspondence driven separately by main subject effects, interaction subject effects, and residual terms, we varied the cross-modality correlation of each of these three sources independently:

$$\rho_{\beta,\beta} \in [-0.5, 0.5], \qquad \rho_{\omega,\omega} \in [-0.5, 0.5], \qquad \rho_{\epsilon,\epsilon} \in [-0.2, 0.2],$$

with

$$\lambda \in \{0.5, 1.0, 2.0\}, \quad \text{and} \quad S = 100.$$

For each parameter combination $(\rho_{\beta,\beta}, \rho_{\omega,\omega}, \rho_{\epsilon,\epsilon}, \lambda)$, we generated 500 independent simulation replicates, each consisting of $S = 100$ subjects' X and Y brain maps.

For replicate ℓ, the interaction subject effects were generated as

$$\begin{pmatrix} \omega_X^{\ell,s} \\ \omega_Y^{\ell,s} \end{pmatrix} \overset{i.i.d.}{\sim} N\left[\begin{pmatrix} 0 \\ 0 \end{pmatrix}, \begin{pmatrix} 1 & \rho_{\omega,\omega} \\ \rho_{\omega,\omega} & 1 \end{pmatrix}\right], \qquad s = 1, \ldots, S.$$

The main subject effects were generated independently across subjects from

$$\begin{pmatrix} \beta_X^{\ell,s} \\ \beta_Y^{\ell,s} \end{pmatrix} \overset{i.i.d.}{\sim} N\left[\begin{pmatrix} 0 \\ 0 \end{pmatrix}, \begin{pmatrix} 1 & \rho_{\beta,\beta} \\ \rho_{\beta,\beta} & 1 \end{pmatrix}\right], \qquad s = 1, \ldots, S.$$

For each brain location v, residual pairs were generated as

$$\begin{pmatrix} \epsilon_{X,v}^{\ell,s} \\ \epsilon_{Y,v}^{\ell,s} \end{pmatrix} \overset{i.i.d.}{\sim} N\left[\begin{pmatrix} 0 \\ 0 \end{pmatrix}, \frac{\mathrm{Var}(\hat{\eta}_{X,v}\ \hat{\omega}_X^s)}{\lambda} \begin{pmatrix} 1 & \rho_{\epsilon,\epsilon} \\ \rho_{\epsilon,\epsilon} & 1 \end{pmatrix}\right], \qquad s = 1, \ldots, S.$$

The simulated brain maps were computed as

$$X_v^{\ell,s} = \hat{\mu}_X + \hat{\alpha}_{X,v} + \beta_X^{\ell,s} + \hat{\eta}_{X,v}\ \omega_X^{\ell,s} + \epsilon_{X,v}^{\ell,s},$$

$$Y_v^{\ell,s} = \hat{\mu}_Y + \hat{\alpha}_{Y,v} + \beta_Y^{\ell,s} + \hat{\eta}_{Y,v}\ \omega_Y^{\ell,s} + \epsilon_{Y,v}^{\ell,s},$$

where $\hat{\mu}_X + \hat{\alpha}_{X,v}$ and $\hat{\mu}_Y + \hat{\alpha}_{Y,v}$ are the group-average cortical thickness and sulcal depth maps, and $\hat{\eta}_{X,v}$ and $\hat{\eta}_{Y,v}$ are empirical estimates of the interaction spatial effects from the HCP cortical thickness and sulcal depth maps.

For each replicate, we applied the SPICE test, the component-specific bootstrap-based tests for H_0^1, H_0^2, and H_0^3, and the overall bootstrap-based method with Bonferroni correction. Main-text results for $\lambda = 2$ are reported in Figure 4. Additional results for $\lambda = 0.5$ and $\lambda = 1.0$ are reported in Supplementary Figures S7–S8.

3 Supplementary Results

3.1 Simulation Comparison Across Different Spatial Resolutions and Autocorrelations

We conducted simulations to evaluate how spatial resolution (the number of locations V) and spatial autocorrelation affect the performances of the spin test, BrainSMASH, and the bootstrap-based method. We repeated simulation studies using the same spatially stationary population-mean brain maps generated as described in Section 2.1.

Effect of Spatial Resolution. Supplementary Figure S5.A illustrates the effect of reduced spatial

resolution (500 parcels; spatial autocorrelation $= 0.5$) on the performances of the three methods. With reduced spatial resolution, both the spin test and BrainSMASH showed zero rejection rates across a wide range of nonzero correlations, indicating severely reduced statistical power. In contrast, the bootstrap-based method maintains the nominal 5% type I error rate at $\rho = 0$ and demonstrates substantially higher power for nonzero ρ, highlighting its robustness to decreases in spatial resolution.

Effect of Spatial Autocorrelation. Supplementary Figure S5.B presents the testing results by the three methods when spatial autocorrelation is increased to 3.0 at the vertex-level resolution. Under this high-autocorrelation setting, both the spin test and BrainSMASH showed zero rejection rates across a markedly wider interval of nonzero correlations, reflecting a further loss of statistical power. In contrast, the bootstrap-based method remains stable, maintaining the nominal 5% type I error rate and exhibiting consistent power for nonzero ρ.

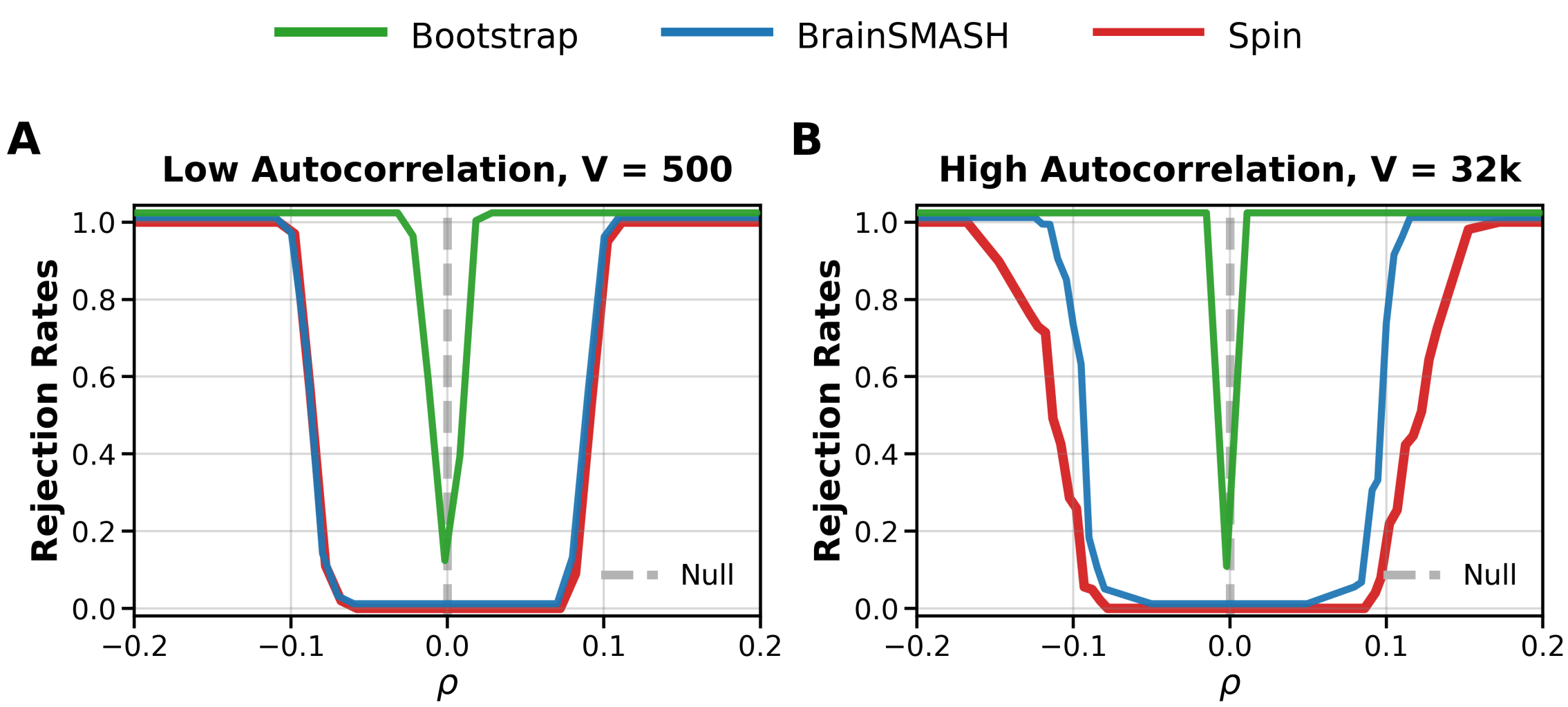

Supplementary Figure S5: Mean rejection rates for stationary population-mean brain maps under different combinations of spatial autocorrelation and spatial resolution. Green, blue, and red curves represent the rejection rates of the bootstrap-based method, BrainSMASH, and the spin test, respectively. A. Low spatial resolution ($V = 500$ parcels) with low spatial autocorrelation (0.5). B. High spatial resolution ($V = 32$k vertices) with high spatial autocorrelation (3.0). The gray dashed line indicates the null hypothesis of zero correspondence. Rejection rates at zero ρ correspond to type I errors, while those for nonzero ρ indicate statistical power.

3.2 Simulation Comparison: Fixed vs. Random Population-Mean Brain Map Assumptions

To demonstrate how different conceptual assumptions about population-mean brain maps lead to distinct inference outcomes, we conducted simulations based on Gaussian random fields (GRFs) with high spatial

autocorrelation ($= 3.0$), following the setup of Burt et al. (2020) and Markello and Misic (2021).

Specifically, we generated 10,000 pairs of population-mean brain maps, $\mu_X^l + \alpha_{X,v}^l$ and $\mu_Y^l + \alpha_{Y,v}^l$, ($l = 1, \ldots, 10,000$), as independent realizations from stationary GRFs with zero expected correspondence between the two modalities. For each pair, we computed the spatial correlation

$$\rho^l = \mathrm{Corr}_{\mathcal{V}}(\mu_X^l + \alpha_{X,v}^l, \mu_Y^l + \alpha_{Y,v}^l).$$

Although the expected correlation across realizations satisfies $\sum_{l=1}^{10,000} \rho^l/10,000 \approx 0$, the strong spatial autocorrelation results in many individual realizations exhibiting nonzero ρ^l values due to chance alignment of spatial patterns.

For each realized pair of population-mean brain maps $\mu_X^l + \alpha_{X,v}^l$ and $\mu_Y^l + \alpha_{Y,v}^l$, we then simulated 500 replicates of $S = 100$ subjects' X and Y brain maps $\{X_v^{l,s}, Y_v^{l,s}, s = 1, \ldots, S\}$ following the procedure described in Section 3.1 of the main text. We applied the spin test, BrainSMASH, and the bootstrap-based method to each replicate and recorded the rejection rates for each corresponding ρ^l.

Supplementary Figure S6 summarizes the rejection rates as a function of the realized correlations ρ^l. When $|\rho^l|$ was large, all three methods, the spin test, BrainSMASH, and the bootstrap-based method, consistently rejected the null hypothesis of zero correspondence. However, for ρ^l values near zero, the spin test and BrainSMASH exhibited near-zero rejection rates, while the bootstrap-based method maintained substantially higher power to detect modest but nonzero correlations.

These findings illustrate the conceptual and inferential consequences of adopting fixed versus random population-mean assumptions. Under the random population-mean assumption, as used in the simulation framework of Markello and Misic (2021), population-mean maps are viewed as random realizations of a spatial process with zero expected correspondence. In this framework, all rejections are considered false positives because, by definition, the underlying process has zero expected correlation.

In contrast, under our fixed population-mean assumption, each realized pair of population-mean maps represents the true, fixed spatial structure of the population. Therefore, a nonzero realized correlation ρ^l indicates genuine correspondence between the two brain maps. Rejections by the bootstrap-based method in these cases are interpreted as true positives, correctly identifying fixed, nonzero spatial relationships.

Together, these simulations highlight how fundamentally different conceptual assumptions—treating population-mean brain maps as fixed versus random—lead to divergent interpretations of type I error and power, as well as distinct conclusions about the validity and conservatism of permutation-based inference.

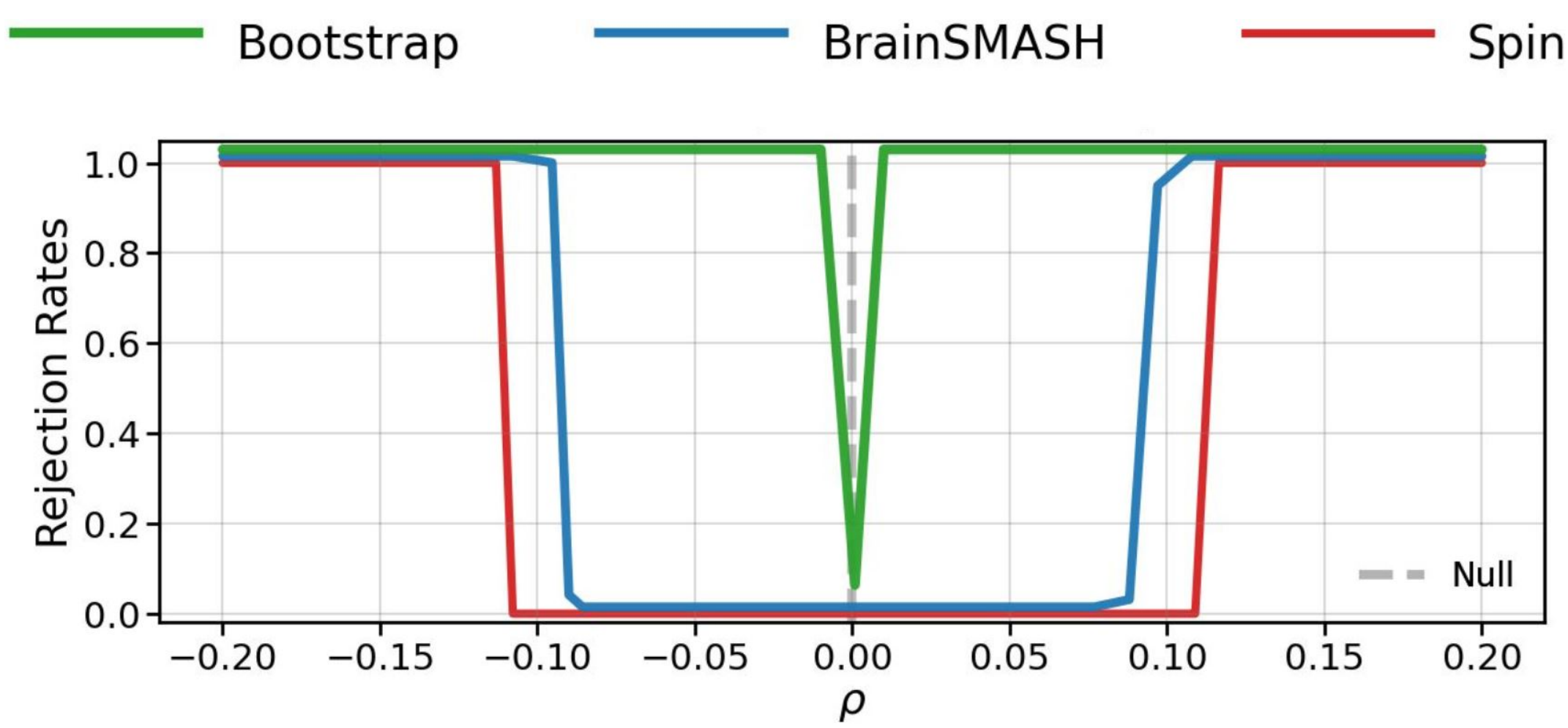

Supplementary Figure S6: Mean rejection rates in simulation studies assessing correspondence between fixed population-mean brain maps with $S = 100$ subjects. The population-mean maps were generated from Gaussian random fields (GRFs) with strong spatial autocorrelation 3.0. Red, blue, and green curves represent mean rejection rates of the spin test, BrainSMASH, and the bootstrap-based method, respectively. The gray dashed line indicates the null hypothesis of zero spatial correlation. Rejection rates at zero ρ correspond to type I errors, while those for nonzero ρ indicate statistical power.

3.3 Simulation Comparison with the SPICE test at Lower SNRs

We extended the simulation studies described in Section 3.3 of the main text to further compare the performance of the bootstrap-based method and the SPICE test under lower signal-to-noise ratios (SNRs), specifically $\lambda = 0.5$ (Supplementary Figure S8) and $\lambda = 1.0$ (Supplementary Figure S7), while keeping the sample size fixed at $S = 100$. These figures present the rejection rates as $\rho_{\beta,\beta}$, $\rho_{\omega,\omega}$, and $\rho_{\epsilon,\epsilon}$ were varied independently. The observed patterns are highly consistent with those in the main text, indicating that the overall performance trends of both methods are stable across SNR levels. Together, these results confirm that the conclusions drawn in the main text remain valid even under lower SNRs: the bootstrap-based method continues to exhibit high statistical power, particularly when correspondence arises from subject-level effects ($\rho_{\beta,\beta}$ and $\rho_{\omega,\omega}$), whereas the SPICE test remains primarily sensitive to correspondence driven by residual correlations ($\rho_{\epsilon,\epsilon}$).

3.4 Bootstrap Distributions

Supplementary Figure S9 presents the bootstrap sampling distributions for estimated correlations ($\hat{\rho}$) between population-mean cortical thickness and sulcal depth brain maps, and estimated correlations between subject-specific random components, $\hat{\rho}_{\beta,\beta}$, $\hat{\rho}_{\omega,\omega}$, $\hat{\rho}_{\epsilon,\epsilon}$, $\hat{\rho}_{\omega,\epsilon}$, and $\hat{\rho}_{\epsilon,\omega}$, across the two modalities.

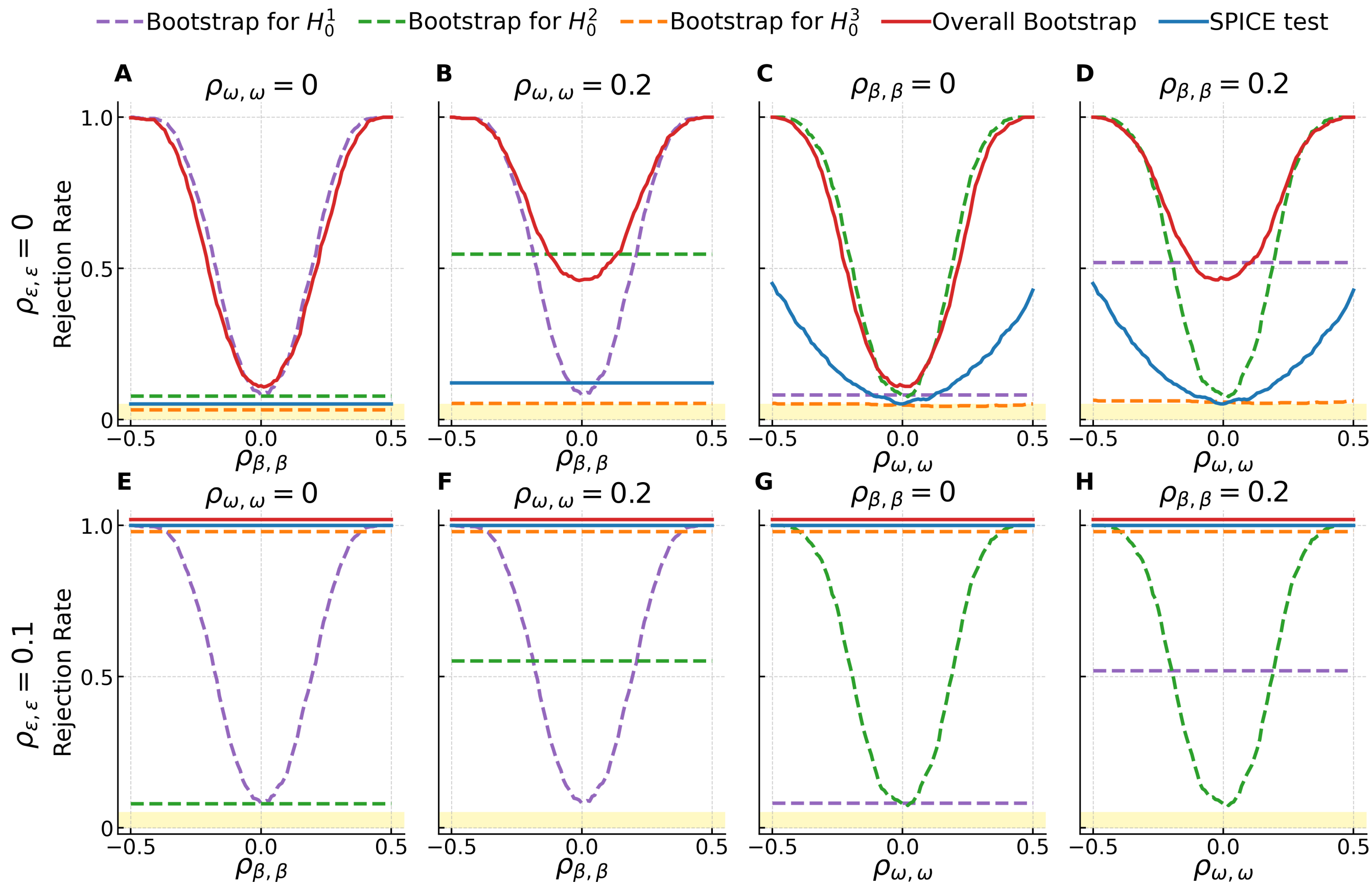

Supplementary Figure S7: Rejection rates of the bootstrap-based method and the SPICE test in simulation studies assessing correspondence between subject-specific brain maps. The correlations $\rho_{\beta,\beta}$, $\rho_{\omega,\omega}$, and $\rho_{\epsilon,\epsilon}$ were varied independently, with SNR $\lambda = 1.0$ and sample size $S = 100$. A-D. Rejection rates across different combinations of $\rho_{\beta,\beta}$ and $\rho_{\omega,\omega}$ with $\rho_{\epsilon,\epsilon} = 0$. E-H. Rejection rates across different combinations of $\rho_{\beta,\beta}$ and $\rho_{\omega,\omega}$ with $\rho_{\epsilon,\epsilon} = 0.1$. Purple, green, and orange curves represent rejection rates of the bootstrap-based method for separately testing three hypotheses: independence between main subject effects ($H_0^1 : \beta_X^s \perp \beta_Y^s$), independence between interaction subject effects ($H_0^2 : \omega_X^s \perp \omega_Y^s$), and independence between residual terms ($H_0^3 : \epsilon_{X,v}^s \perp \epsilon_{Y,v}^s$). Red and blue curves represent rejection rates of the overall bootstrap-based method and the SPICE test, respectively. The yellow-shaded region denotes the 5% significance threshold.

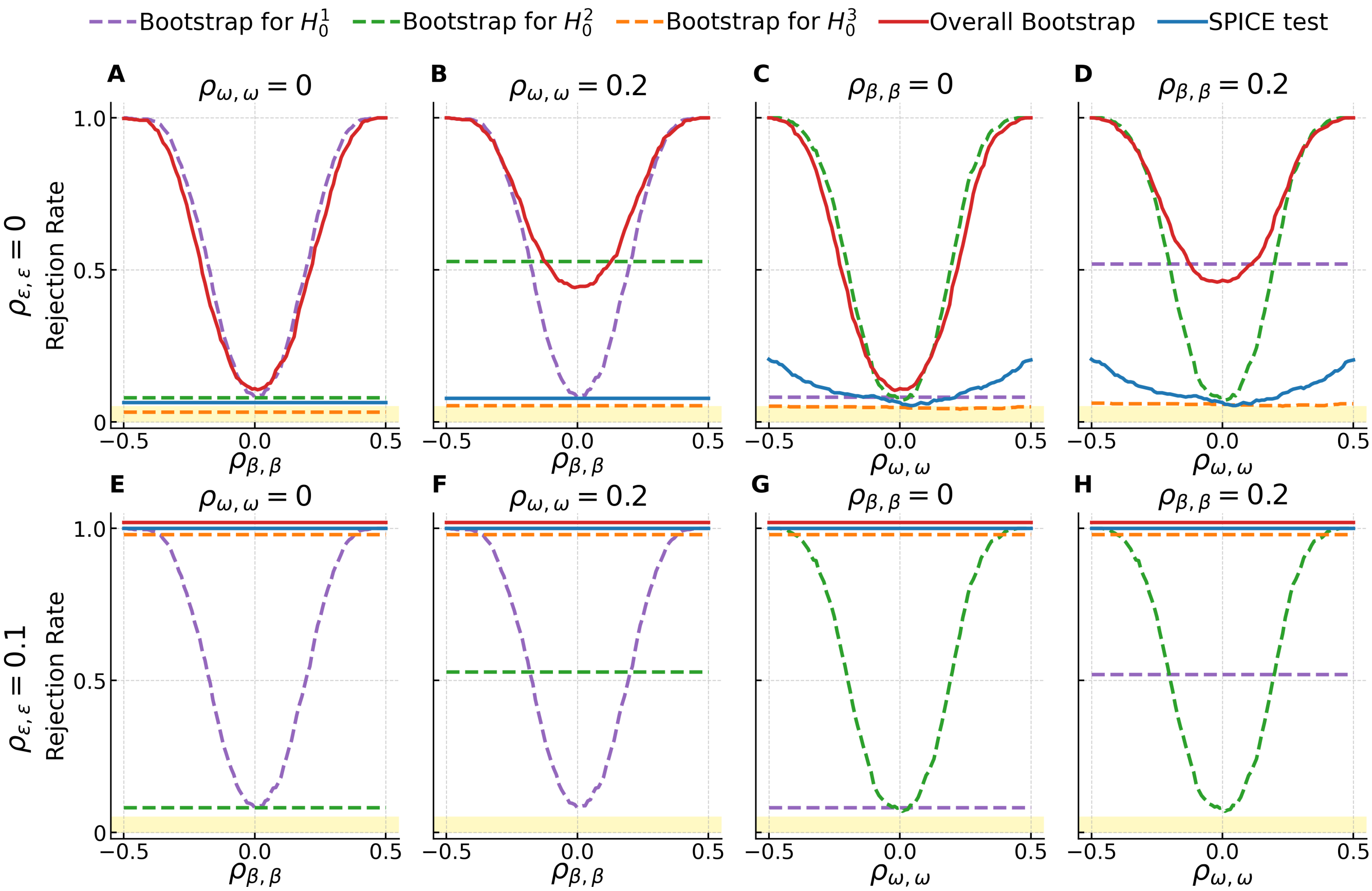

Supplementary Figure S8: Rejection rates of the bootstrap-based method and the SPICE test in simulation studies assessing correspondence between subject-specific brain maps. The correlations $\rho_{\beta,\beta}$, $\rho_{\omega,\omega}$, and $\rho_{\epsilon,\epsilon}$ were varied independently, with SNR $\lambda = 0.5$ and sample size $S = 100$. A-D. Rejection rates across different combinations of $\rho_{\beta,\beta}$ and $\rho_{\omega,\omega}$ with $\rho_{\epsilon,\epsilon} = 0$. E-H. Rejection rates across different combinations of $\rho_{\beta,\beta}$ and $\rho_{\omega,\omega}$ with $\rho_{\epsilon,\epsilon} = 0.1$. Purple, green, and orange curves represent rejection rates of the bootstrap-based method for separately testing three hypotheses: independence between main subject effects ($H_0^1 : \beta_X^s \perp \beta_Y^s$), independence between interaction subject effects ($H_0^2 : \omega_X^s \perp \omega_Y^s$), and independence between residual terms ($H_0^3 : \epsilon_{X,v}^s \perp \epsilon_{Y,v}^s$). Red and blue curves represent rejection rates of the overall bootstrap-based method and the SPICE test, respectively. The yellow-shaded region denotes the 5% significance threshold.

Similarly, Supplementary Figure S10 shows the corresponding bootstrap sampling distributions for the correlations between motor and language contrast brain maps.

These figures illustrate the empirical sampling variability of each estimated correspondence measure and show the 95% bootstrap confidence intervals (CIs) related to the inference results summarized in the population-mean and subject-specific correspondence tables in the main text.

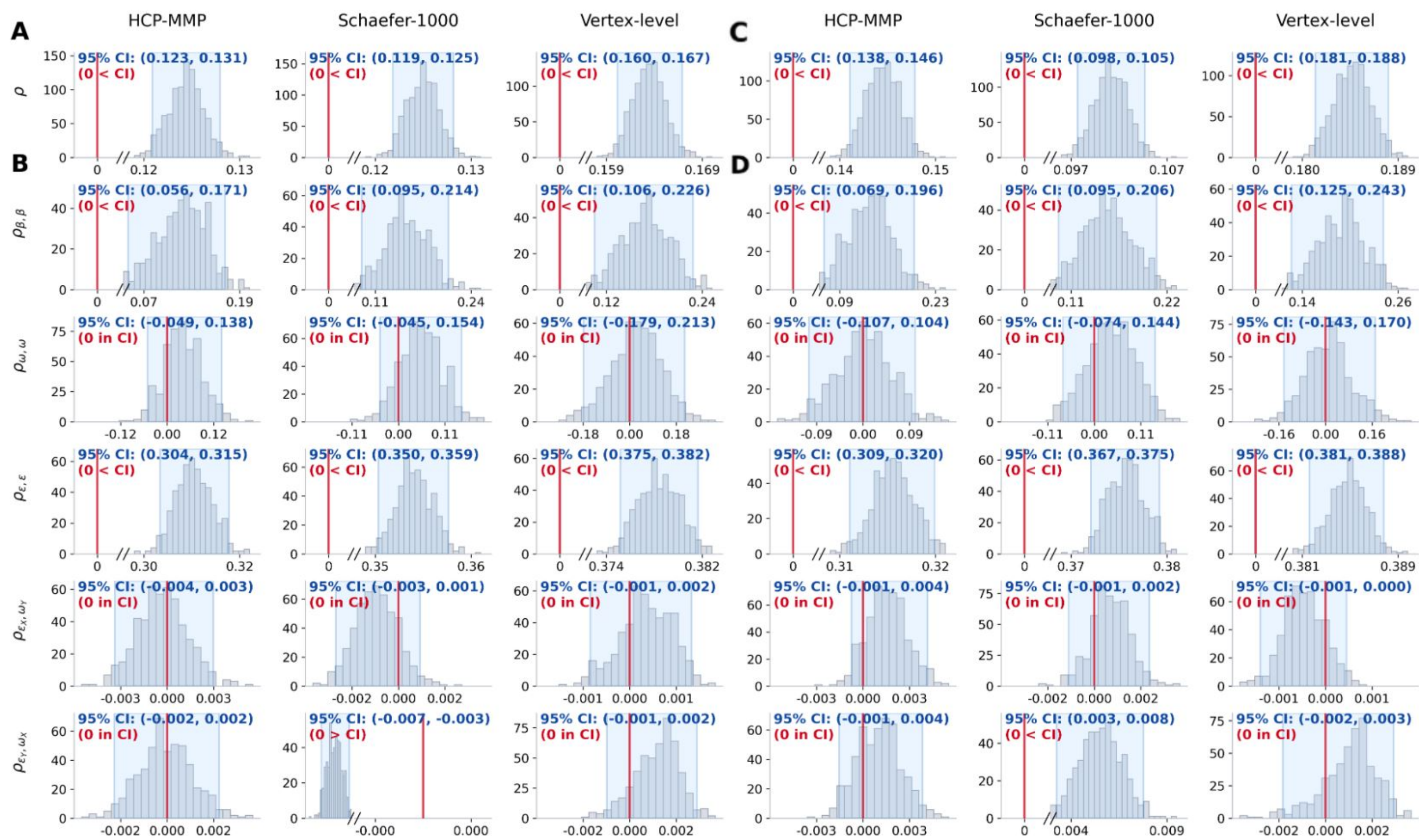

Supplementary Figure S9: Bootstrap sampling distributions for assessing correspondence between cortical thickness and sulcal depth brain maps in the left (A, B) and right (C, D) hemispheres. Each column corresponds to one of three spatial resolutions. A and C, Bootstrap sampling distributions for the estimated correlations between population-mean brain maps, $\hat{\rho}$. B and D. Bootstrap sampling distributions for the estimated correlations between subject-specific random components, $\hat{\rho}_{\beta,\beta}$, $\hat{\rho}_{\omega,\omega}$, $\hat{\rho}_{\epsilon,\epsilon}$, $\hat{\rho}_{\omega,\epsilon}$, and $\hat{\rho}_{\epsilon,\omega}$. Each subfigure shows the bootstrap distribution (gray), with the 95% confidence interval (blue band) and the null-hypothesis value (red line). Axis breaks indicate omitted intervals when confidence intervals are far from the null value.

3.5 Empirical Covariance and Component Attribution

To characterize spatial dependence across the cortex, we computed empirical across-subject covariance matrices between Schaefer-1000 parcels, separately by hemisphere and modality. Supplementary Figure S11 displays these covariance heatmaps for cortical thickness, sulcal depth, language contrast, and

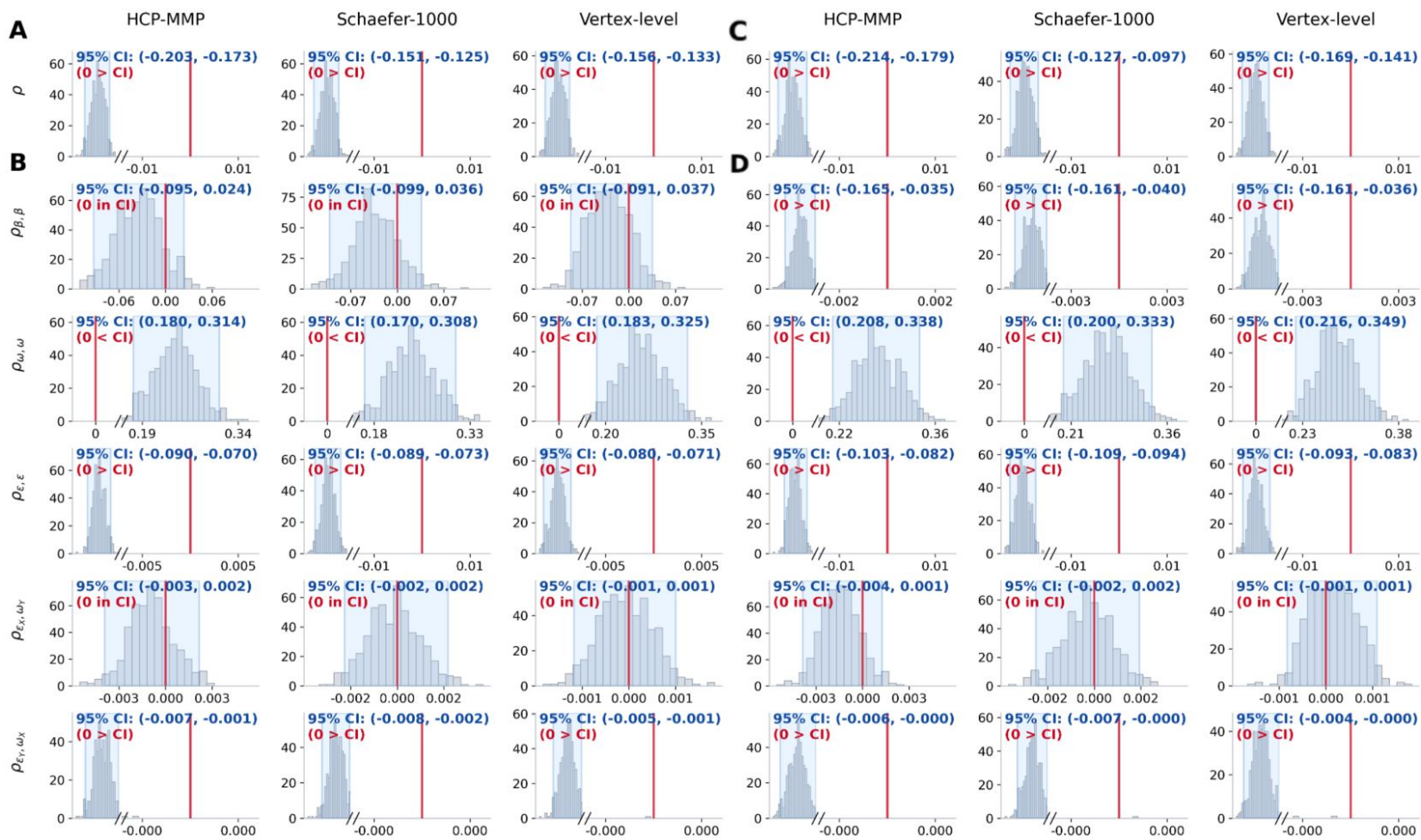

Supplementary Figure S10: Bootstrap sampling distributions for assessing correspondence between motor contrast and language contrast brain maps in the left (A, B) and right (C, D) hemispheres. Each column corresponds to one of three spatial resolutions. A and C, Bootstrap sampling distributions for the estimated correlations between population-mean brain maps, $\hat{\rho}$. B and D. Bootstrap sampling distributions for the estimated correlations between subject-specific random components, $\hat{\rho}_{\beta,\beta}$, $\hat{\rho}_{\omega,\omega}$, $\hat{\rho}_{\epsilon,\epsilon}$, $\hat{\rho}_{\omega,\epsilon}$, and $\hat{\rho}_{\epsilon,\omega}$. Each subfigure shows the bootstrap distribution (gray), with the 95% confidence interval (blue band) and the null-hypothesis value (red line). Axis breaks indicate omitted intervals when confidence intervals are far from the null value.

motor contrast in both the left and right hemispheres. The block-like, modality-specific patterns indicate spatially varying dependence across the cortex, motivating flexible Gaussian process models that can capture these complex and localized structures.

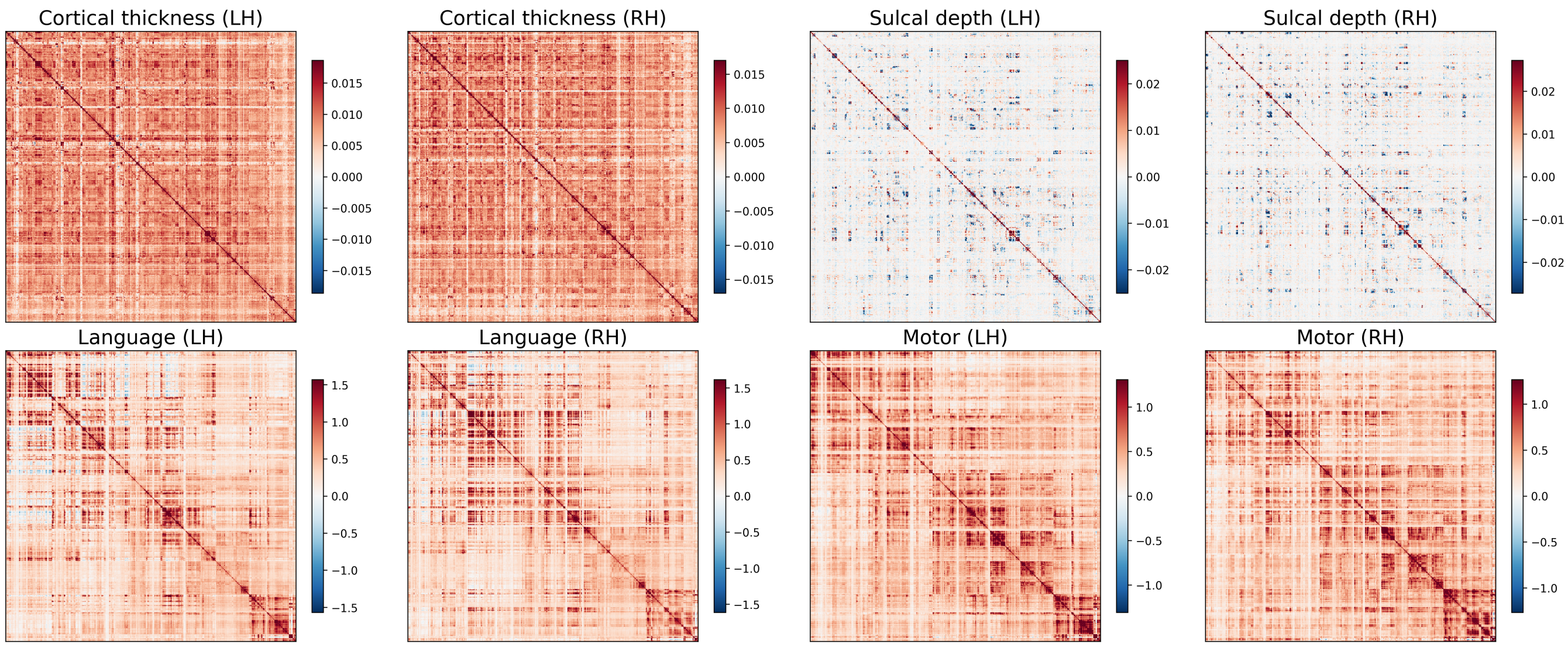

Supplementary Figure S11: Heatmaps of covariance matrices for structural (cortical thickness and sulcal depth) and functional (language and motor contrast) brain maps. Both the left hemisphere (LH) and right hemisphere (RH) in the Schaefer-1000 parcellation are shown; region ordering follows the Schaefer-1000 parcellation.

To assess whether residual variation is related to subject-level effects, we computed the across-subject correlation between fitted residuals and the estimated main subject effect at each Schaefer-1000 parcel, separately for each modality. Supplementary Figure S12 displays these associations on the cortical surface. The spatial heterogeneity of these maps indicates that residual associations with subject-level effects can vary across cortical regions.

3.6 Correlations Between Interaction Effects and Residual Terms

Supplementary Table 1 summarizes the bootstrap-based inference results for correlations between subject-level interaction effects and residual terms ($\rho_{\epsilon,\omega}$ and $\rho_{\omega,\epsilon}$) in both structural and functional brain maps. Panel (A) reports results for cortical thickness and sulcal depth, while Panel (B) reports results for the motor and language contrast maps.

Across all spatial resolutions and hemispheres, and for both structural and functional brain maps, the 95% confidence intervals for these cross-term correlations were tightly concentrated around zero, with all interval endpoints between -0.008 and 0.008. Several intervals excluded zero, indicating statistically

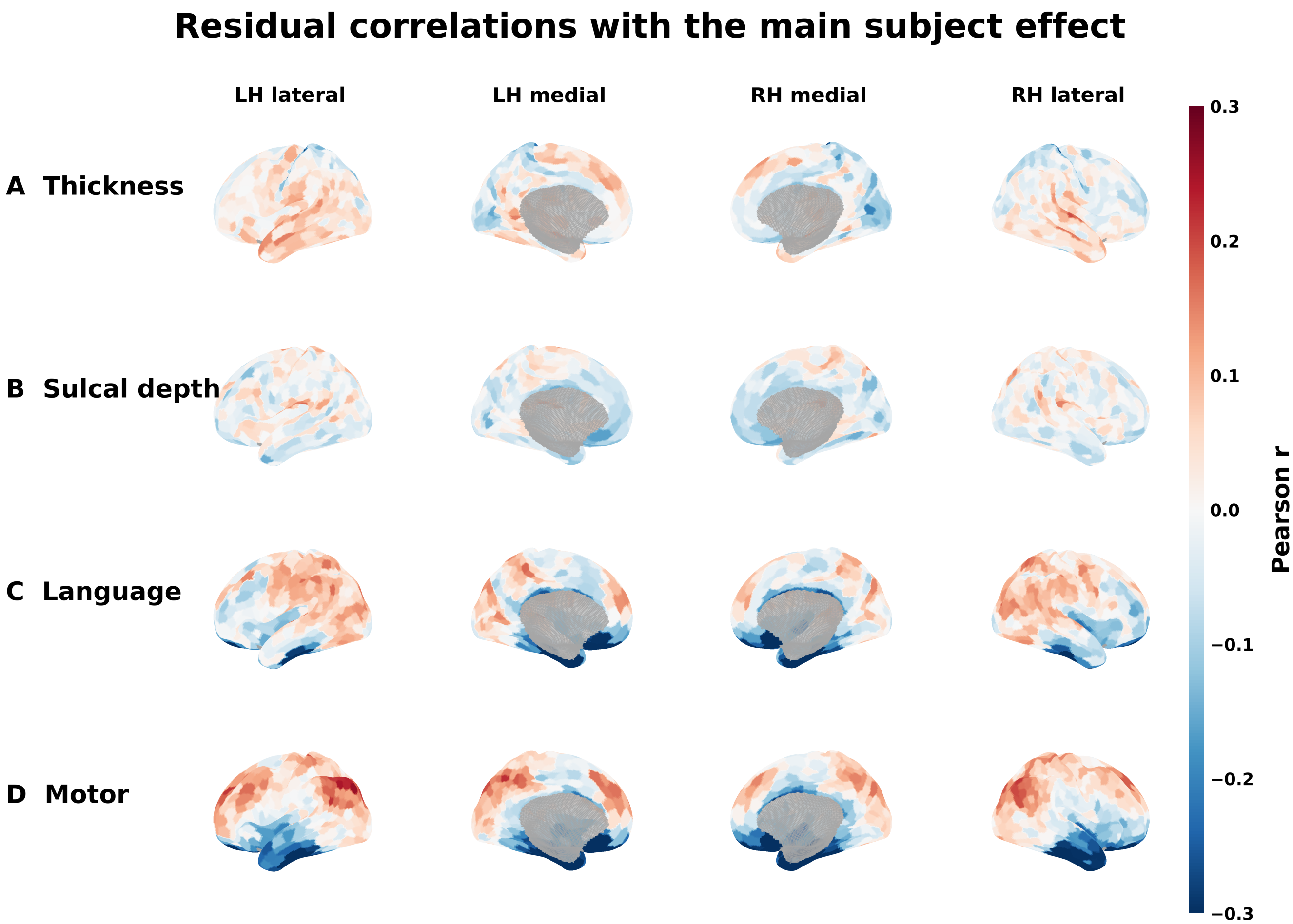

Supplementary Figure S12: Cortical maps of residual associations with the main subject effect. Colors indicate the across-subject association between fitted residuals and the estimated main subject effect for cortical thickness, sulcal depth, language contrast, and motor contrast. Lateral and medial views are shown for both hemispheres.

detectable cross-component associations in some settings. However, their magnitudes were extremely small, suggesting that dependencies between subject-level interaction effects and residual variability make little practical contribution to the observed correspondence.

Supplementary Table 1: Bootstrap-based inference results for correlations between interaction subject effects and residual terms in (A) cortical thickness and sulcal depth brain maps and (B) motor contrast and language contrast brain maps.

Atlas	Hem.	Bootstrap-Based Cross-Term Tests			
		$\rho_{\epsilon,\omega}$ (H_0^4)		$\rho_{\omega,\epsilon}$ (H_0^5)	
		95% CI	Decision	95% CI	Decision
**(A) Cortical Thickness–Sulcal Depth**					
HCP-MMP (180 parcels)	L	(-0.004, 0.003)	FTR	(-0.002, 0.002)	FTR
	R	(-0.001, 0.004)	FTR	(-0.001, 0.004)	FTR
Schaefer-1000 (500 parcels)	L	(-0.003, 0.001)	FTR	(-0.007, -0.003)	Reject
	R	(-0.001, 0.002)	FTR	(0.003, 0.008)	Reject
Vertex-level (32k vertices)	L	(-0.001, 0.002)	FTR	(-0.001, 0.002)	FTR
	R	(-0.001, 0.001)	FTR	(-0.002, 0.003)	FTR
**(B) Motor Contrast–Language Contrast**					
HCP-MMP (180 parcels)	L	(-0.003, 0.002)	FTR	(-0.007, -0.001)	Reject
	R	(-0.004, 0.001)	FTR	(-0.006, -0.001)	Reject
Schaefer-1000 (500 parcels)	L	(-0.002, 0.002)	FTR	(-0.008, -0.002)	Reject
	R	(-0.002, 0.002)	FTR	(-0.007, -0.001)	Reject
Vertex-level (32k vertices)	L	(-0.001, 0.001)	FTR	(-0.005, -0.001)	Reject
	R	(-0.001, 0.001)	FTR	(-0.004, -0.001)	Reject

FTR = Fail to Reject. Decision indicates whether the null hypothesis is rejected (95% CI excludes zero).

3.7 Hemispheric Difference in Functional Main Subject Effects

To assess hemispheric differences in functional subject-level correspondence, we compared the main subject-effect correlations between motor and language contrast maps across hemispheres. The difference was defined as $\Delta = \rho^L_{\beta,\beta} - \rho^R_{\beta,\beta}$, where $\rho^L_{\beta,\beta}$ and $\rho^R_{\beta,\beta}$ denote the left- and right-hemisphere correlations, respectively. Because the motor–language correlations were negative, positive values of Δ indicate a stronger negative association in the right hemisphere.

Supplementary Table 2: Bootstrap-based confidence intervals for the hemispheric difference in main subject-effect correlations between motor and language contrast maps.

Atlas / Resolution	95% CI for $\Delta = \rho^{L}_{\beta,\beta} - \rho^{R}_{\beta,\beta}$
HCP-MMP (360 parcels)	$(0.02,\ 0.12)$
Schaefer-1000 (1000 parcels)	$(0.02,\ 0.11)$
Vertex-level (fs_LR 32k)	$(0.02,\ 0.12)$

3.8 Calibration Under Residual Spatial Dependence

The proposed nonparametric bootstrap procedure does not require distributional assumptions on the residuals or estimation of their spatial covariance structure. To assess whether local residual dependence affects calibration, we conducted a simulation study on the `fs_LR 32k` cortical surface. Residuals were generated from Gaussian random fields with autocorrelation parameters $\alpha_{\mathrm{GRF}} \in \{0, 1.5, 3.0\}$, representing independent, mildly autocorrelated, and strongly autocorrelated residuals. Data were generated under the null hypothesis of zero cross-map correspondence, and the bootstrap-based procedure was applied at the nominal 5% significance level.

Supplementary Table 3 shows that empirical false-positive rates remained close to 5% across all levels of residual spatial autocorrelation. These results indicate that the proposed bootstrap procedure maintains valid calibration even when residuals exhibit strong local spatial dependence.

Supplementary Table 3: Empirical false-positive rates under the null hypotheses on the `fs_LR 32k` cortical surface for different levels of residual spatial autocorrelation.

Residual Spatial Dependence	H_0 (α)	H_0^1 (β)	H_0^2 (ω)	H_0^3 (ϵ)
$\alpha_{\mathrm{GRF}} = 0$ (independent)	0.05	0.06	0.05	0.06
$\alpha_{\mathrm{GRF}} = 1.5$ (mild)	0.07	0.07	0.06	0.07
$\alpha_{\mathrm{GRF}} = 3.0$ (strong)	0.06	0.06	0.06	0.07

References

Burt, J. B., Helmer, M., Shinn, M., Anticevic, A., & Murray, J. D. (2020). Generative modeling of brain maps with spatial autocorrelation. *NeuroImage*, *220*, 117038.

Markello, R. D., & Misic, B. (2021). Comparing spatial null models for brain maps. *NeuroImage*, *236*, 118052.

Viladomat, J., Mazumder, R., McInturff, A., McCauley, D. J., & Hastie, T. (2014). Assessing the significance of global and local correlations under spatial autocorrelation: A nonparametric approach. *Biometrics*, *70*(2), 409–418.
